# Advances in neuroproteomics for neurotrauma: unraveling insights for personalized medicine and future prospects

**DOI:** 10.3389/fneur.2023.1288740

**Published:** 2023-11-22

**Authors:** Firas Kobeissy, Mona Goli, Hamad Yadikar, Zaynab Shakkour, Milin Kurup, Muhammad Ali Haidar, Shahad Alroumi, Stefania Mondello, Kevin K. Wang, Yehia Mechref

**Affiliations:** ^1^Department of Neurobiology, School of Medicine, Neuroscience Institute, Atlanta, GA, United States; ^2^Department of Chemistry and Biochemistry, Texas Tech University, Lubbock, TX, United States; ^3^Department of Biological Sciences Faculty of Science, Kuwait University, Safat, Kuwait; ^4^Department of Pathology and Anatomical Sciences, University of Missouri School of Medicine, Columbia, MO, United States; ^5^Alabama College of Osteopathic Medicine, Dothan, AL, United States; ^6^Division of Neurobiology, Free University of Berlin, Berlin, Germany; ^7^Department of Biomedical and Dental Sciences and Morphofunctional Imaging, University of Messina, Messina, Italy

**Keywords:** proteomics, neuroproteomics, neurotrauma, personalized medicine (PM), traumatic brain injuries (TBI), artificial intelligence (AI), machine learning (ML)

## Abstract

Neuroproteomics, an emerging field at the intersection of neuroscience and proteomics, has garnered significant attention in the context of neurotrauma research. Neuroproteomics involves the quantitative and qualitative analysis of nervous system components, essential for understanding the dynamic events involved in the vast areas of neuroscience, including, but not limited to, neuropsychiatric disorders, neurodegenerative disorders, mental illness, traumatic brain injury, chronic traumatic encephalopathy, and other neurodegenerative diseases. With advancements in mass spectrometry coupled with bioinformatics and systems biology, neuroproteomics has led to the development of innovative techniques such as microproteomics, single-cell proteomics, and imaging mass spectrometry, which have significantly impacted neuronal biomarker research. By analyzing the complex protein interactions and alterations that occur in the injured brain, neuroproteomics provides valuable insights into the pathophysiological mechanisms underlying neurotrauma. This review explores how such insights can be harnessed to advance personalized medicine (PM) approaches, tailoring treatments based on individual patient profiles. Additionally, we highlight the potential future prospects of neuroproteomics, such as identifying novel biomarkers and developing targeted therapies by employing artificial intelligence (AI) and machine learning (ML). By shedding light on neurotrauma’s current state and future directions, this review aims to stimulate further research and collaboration in this promising and transformative field.

## Highlights

- Neuroproteomics represents a new arrow to the precision medicine bow, allowing us to characterize neurological disorders more precisely and tailor medical treatments to specific individual patient needs.
- Combination of neuroproteomics with novel powerful computational tools and artificial intelligence can address the highly complex networks that underlie pathobiological mechanisms of brain injury.

## 1 Introduction

### 1.1 General overview of neuroproteomics

Several platforms have been used for decades to carry out protein-specific research. Proteomics has been introduced to study the proteome of a given biological system’s expression, interaction, functions, and modifications (1). Among the primary endeavors of proteomics include identifying and discovering new molecular protein hits that can indicate a specific homeostatic state. Over its development, proteomics has become an optimal approach for accurate diagnostic and prognostic technology, reflected in the logarithmic advancement in this field and its technological applications (2–5).

The vast history of neuroproteomics has developed over a long period, dating back to the initial development of genome and genetic studies. The human genome project paved the way for initiating thes human proteome project. This project explored the human proteome’s many biological and functional properties associated with approximately 20,300 protein-coding genes (6). As such, this allowed for studying the role of these gene-coded proteins in both healthy and pathological conditions using proteomics, systems biology, and bioinformatics tools (7). These previous projects highlighted the proteome’s complexity, where a single gene can translate into several protein isoforms (2, 8). Several factors contributing to this diversity in protein isoforms include alternative splicing and post-translational modifications (PTMs).

The modern field of proteomics is complementary to the genomics field. Proteomics represents a downstream transition of the genome map and has been used to evaluate the biological system’s genotype signature (8). By validating the translation of its proposed altered genomic map and assessing the phenotypic output, we can link the genome with the proteome. Nevertheless, this association may be affected by several confounding factors, such as different physiological compartments, conditions, PTMs, and other external factors, thereby leading to different protein structures and chemical isoforms (2).

Proteomics studies are conducted *in-vivo* and *in-vitro* through various approaches and can create models for multiple conditions related to protein concentration levels and structure modifications. PTMs are crucial for the characterization and analysis of numerous diseases, including neurodegenerative disorders (9).

Ultimately, proteomics’ development spans a vast history and has developed a new and more efficient technique for protein identification. Today, proteomics research and technological advancements can be paired together to advance precision medicine and clinical applications. With the introduction of Artificial Intelligence (AI) and Machine Learning (ML), technology can organize a more accurate form of personalized medicine, providing the most accurate implications for individualized and unique treatment options for every patient (10). This review will discuss several aspects of proteomics and link its potential role in technology-based personalized medicine.

Neuroproteomics is a field that studies the nervous system to understand disorders like neuropsychiatric, degenerative disorders, and neurotrauma-related injuries (11–13), i.e., traumatic brain injuries (TBI) (14), spinal cord injury (SCI) (15), and stroke (16). It is classified into four categories: Expression Neuroproteomics, Functional Neuroproteomics, Clinical Neuroproteomics, and Neuroproteomics Informatics (2). The first category focuses on profiling the proteome, the second one investigates the functional properties of individual sets of proteins (9), the third aims at discovering drugs and novel biomarkers for pathological conditions (17, 18), and the fourth is dedicated to computational tools and specific databases enabling the analysis of proteomics data sets (19–22). With information from all four categories, a neuroscientist can propose new algorithms and faster outputs to conduct clinical prognosis and diagnosis.

In the neuroproteomics fields’ development and advancements, some significant challenges arise from the CNS (23). Analyzing the CNS is difficult due to the presence of over 20,000 proteins in the brain that are differentially expressed within different regions. Overall, it becomes incredibly challenging to comprehensively study the brain proteome and its dynamic function without using high-resolution protein identification and separation techniques. Neuroproteomics composition analysis challenges have arisen with the neural complexity created by the network structure of axons, dendrites, and synapses. Challenges in neuroproteomics composition include difficulty analyzing different brain regions due to limitations in the number of samples to be obtained for analysis (24). Therefore, identifying proteins that are expressed in small quantities is exceptionally challenging. Technological advancements, clinical trials, and precision tools have been developed to combat these challenges in the neuroproteomics (25).

In continuation, due to the nonlinear relation between the genome and the proteome, it is challenging to draw a direct correlation and association between mRNA expression and protein translation (2, 3, 26–30). This is attributable to different factors, including alternative splicing, which is highly frequent in brain tissue, generating thousands of copies of positively related splices from a single gene. For example, the protein Cadherin has 18 different isoforms linked to morphogenic and functional roles in developing the nervous system (27, 30–32). An average of 10 protein isoforms can be generated within a single gene, owing to the proteome’s complexity compared to the genome (33, 34). This complexity is amplified by the numerous dynamic PTMs, which can reach around 400 possible modifications (27, 35).

Mass spectrometry (MS)-based proteomics has proven to be an indispensable tool for molecular and cellular biology, as well as for the emerging field of system biology (36). It has been successful in a variety of applications, including studying protein–protein interactions, mapping organelles, and generating quantitative protein profiles from diverse species (36). With its ability to identify and quantify thousands of proteins from complex samples, MS is expected to have a significant impact on the fields of biology and medicine (36). Classic approaches in proteomics use MS coupled with advanced separation techniques to analyze protein interactions and structures (37–39). MS-based techniques involve bottom-up or top-down analysis (40). In the former analysis, proteins undergo enzymatic digestion first, followed by fragment identification via shotgun-proteomics methods that involve nanoflow liquid chromatography (nanoLC) (41). However, the latter goes without enzymatic digestion, where the entire intact protein undergoes analysis. Interestingly, other tagging techniques have been coupled to MS, such as isobaric tags for relative and absolute quantitation (iTRAQ) (42) and stable isotope labeling with amino acids in cell culture (SILAC) (43). These allow for proteomics changes and PTM assessment analyzes as with phosphorylation-dependent activation. T.

**Figure 1:**
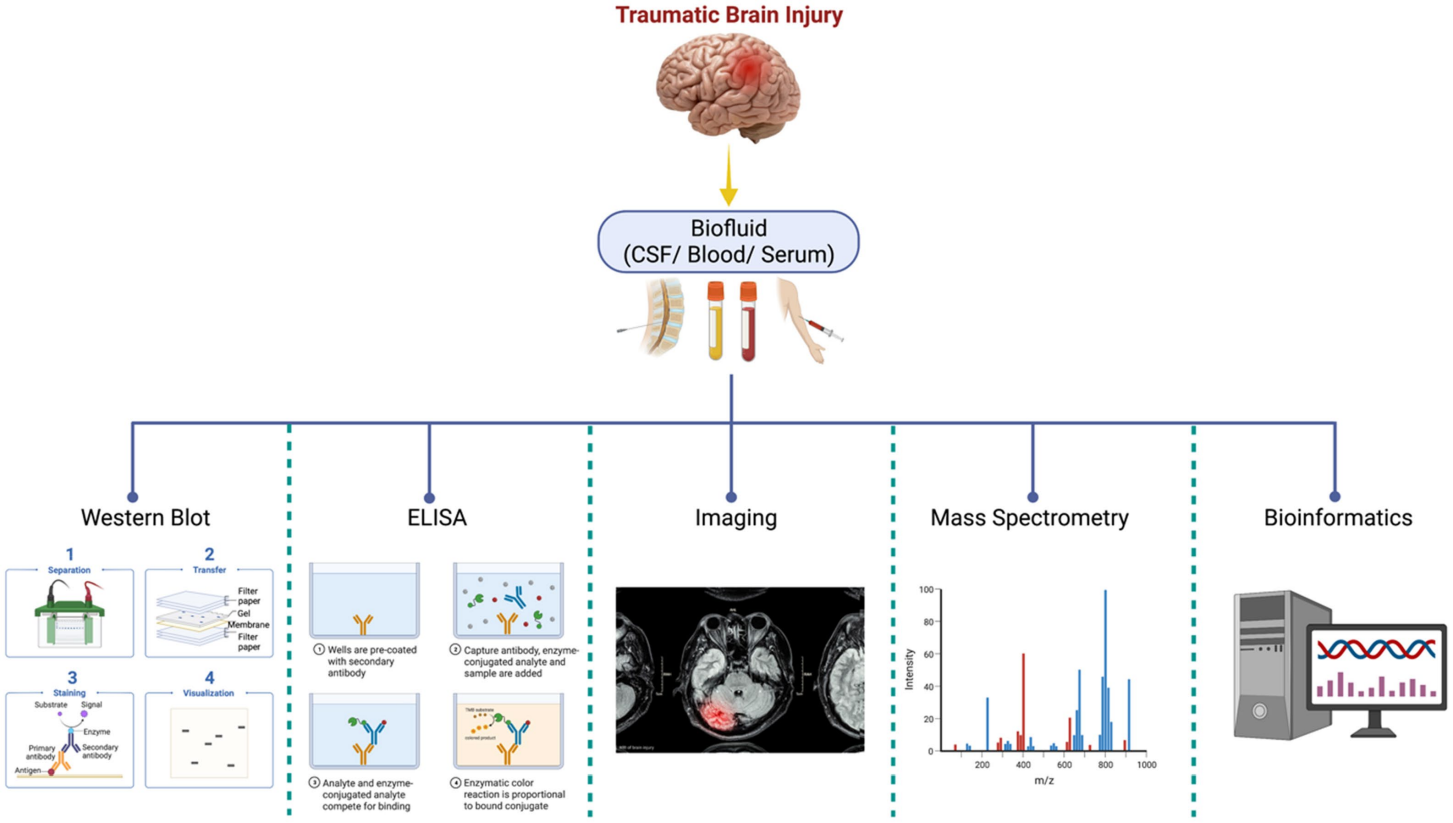
Neuroproteomics quantification methodologies. Neuroproteomics utilizes many molecular techniques to quantify and characterize protein concentration and quantity. SDS-Page Gel Separation (Western Blot), ELISA, Mass Spectrometry, Bioinformatic data, and neuroimaging correlations utilize quantitative data collection methods within proteomics.

Another field approach relies on antibody-based techniques without MS and involves targeted biomarker proteins through an antibody panel or array platforms (24, 44). Although this offers high specificity and sensitivity in identifying the proteins, it cannot identify novel protein biomarkers. These technologies include high-throughput immunoblotting (HTPI) (45, 46) and antibody panel/microarray (47). The former utilizes unstable channels where samples can be identified via immunoblotting systems that use PAGE followed by an antibody probing. The latter technology is based on DNA microarrays or ELISA arrays, where pre-labeled proteins in samples with differential fluorescent dyes are probed against an antibody platform. A mixture of qualitative characterization (PTM, disease characterization, injury severity scores) and quantitative techniques (MS, iTRAQ, SILAC, ELISA, immunoglobulin assays). Figure 1 illustrates the general proteomics quantification methodologies. Neuroproteomics utilizes many molecular techniques to quantify and characterize protein concentration and quantity. SDS-Page Gel Separation (Western Blot), ELISA, Mass Spectrometry, Bioinformatic data, and neuroimaging correlations utilize quantitative data collection methods within proteomics.

**Figure 2:**
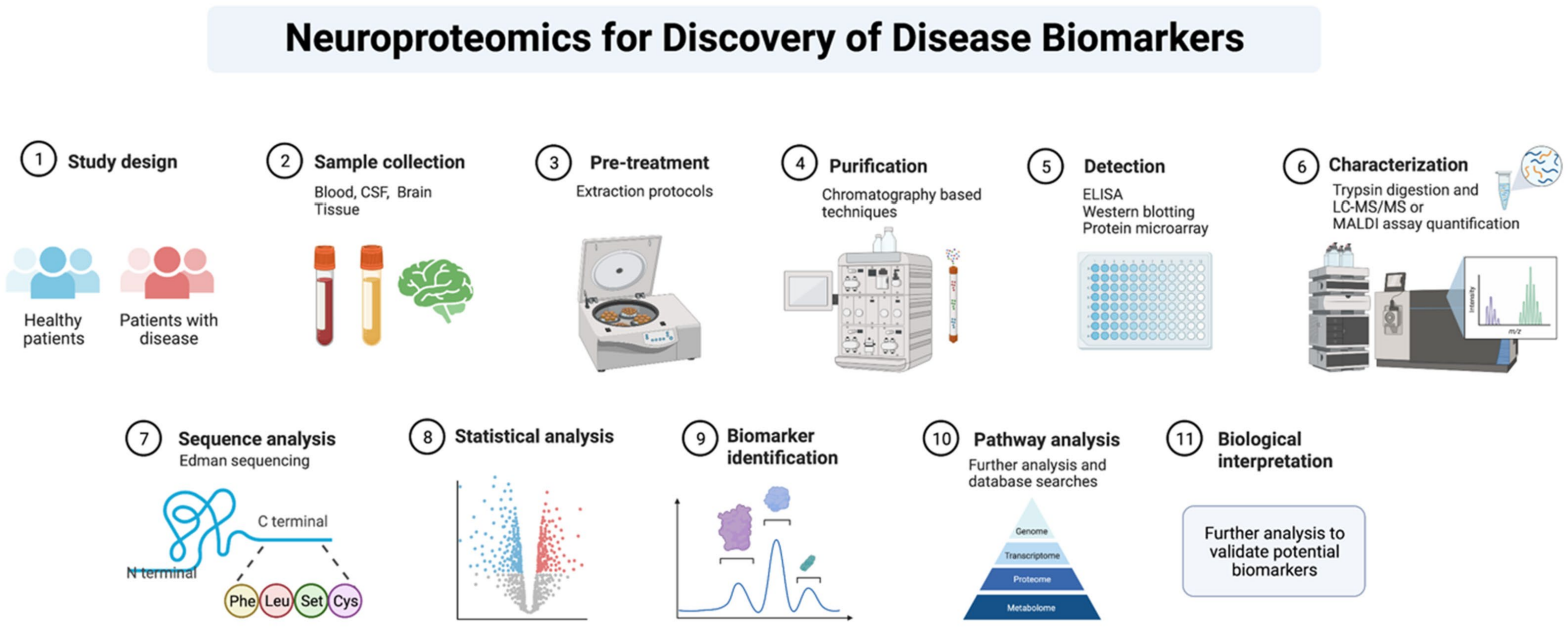
General neuroproteomics workflow for discovery of disease biomarkers.

Neuroproteomics is a process that analyzes the concentration and structure of different biomarkers using specific protocols and methodologies. The process involves extracting biosamples through invasive and non-invasive procedures, collecting biofluids for analysis, and identifying and quantifying proteins. Scientists use various methods, such as SDS-PAGE, Western blots, and mass spectrometry, to identify and quantify the proteins. They also use bioinformatics and machine databases to confirm and classify the quantified proteomics datasets (2, 11). By analyzing potential biomarkers and evaluating their concentration under independent conditions, scientists can pinpoint what is causing neurodegeneration. Neuroproteomics helps neuroscientists diagnose conditions efficiently and identify novel therapeutic targets to develop personalized medicine (48). Figure 2 presents the general neuroproteomics workflow for discovering disease biomarkers.

Importantly, the advancement and adaptation of these technologies can permit the characterize PTM, such as glycosylation. This largely unexplored field has great potential for the identification of novel biomarkers while uncovering their biological and pathobiological role. In the next section, we focus on this specific aspect illustrating proteomics methods and approaches to accurately and reliably identify and profile the brain glycoproteome.

### 1.2 Protein glycosylation

Glycosylation is one of the most predominant post-translational modifications of proteins. As protein sequencing data suggest, the glycosylation rate of mammalian cell proteins is estimated to be greater than 50% (49–51). In the glycosylation process, carbohydrates are added to lipids, proteins, and other organic molecules within or outside the cells (49–51). Glycosylation is a tightly regulated process, as it is a site-specific enzymatic modification (52). A variety of protein properties, such as solubility, are affected by glycans of secreted glycoproteins, whereas cell surface glycosylated proteins have been implicated in various cellular processes, such as cell-to-cell communication (53). The major glycans of glycoproteins are classified into two groups based on their glycan–peptide bonds, namely the N-glycan and O-glycan. The amino sugar N-acetylglucosamine (GlcNAc) is linked to the asparagine amide group to form N-glycans, whereas O-glycans are linked to the hydroxyl groups of the serine and threonine amino acids of polypeptides by N-acetylgalactosamine (49–51, 54).

Aberrant protein glycosylation has been linked to many diseases such as Alzheimer’s Disease (AD) (55, 56), TBI (57), Parkinson’s Disease (PD) (58), congenital/metabolic disorders (59, 60), diabetes (61, 62), inflammation (63), bacterial/viral infectious diseases (64–66), and various cancers (67–71). Aside from this, due to structural heterogeneity, the presence of isomeric glycans complicates the structural analysis of glycans and glycoproteins (72). It is also known that structural heterogeneity affects the biological roles of these glycans/glycoproteins in various diseases (73). Hence, studying the isomeric forms of glycans and glycopeptides is crucial. The importance of proteins and glycoproteins in biological processes, as well as the correlation between their altered expression and a wide range of diseases, makes proteomics and glycoproteomics promising frontiers in the development of biomarkers (74). These biomarkers offer unparalleled opportunities for refinement in clinical characterization and improve disease phenotyping. Such improved characterization and stratification will enable more targeted treatments.

As the first step for the characterization of glycosylated proteins, they need to be isolated from complex biological samples that include both glycosylated and nonglycosylated proteins. Upon isolation, glycoproteins/glycopeptides are enriched, digested by proteolysis, and detected/identified via mass spectrometry-based techniques using glycoproteomics platforms, a subset of the proteomics (75). For the purification and separation of glycoproteins, various types of HPLC are available, including ion exchange methods, hydrophobic interactions, size exclusions, and affinity chromatography. It is necessary to develop fast and robust analytical techniques to study the altered glycosylation profiles induced by a specific disease (76). A number of techniques have previously been used to characterize glycoproteins, including lectin affinity, hydrazide chemistry, and peptide or protein enrichment, which involves deglycosylation and other chemical modifications (77, 78).

**Figure 3:**
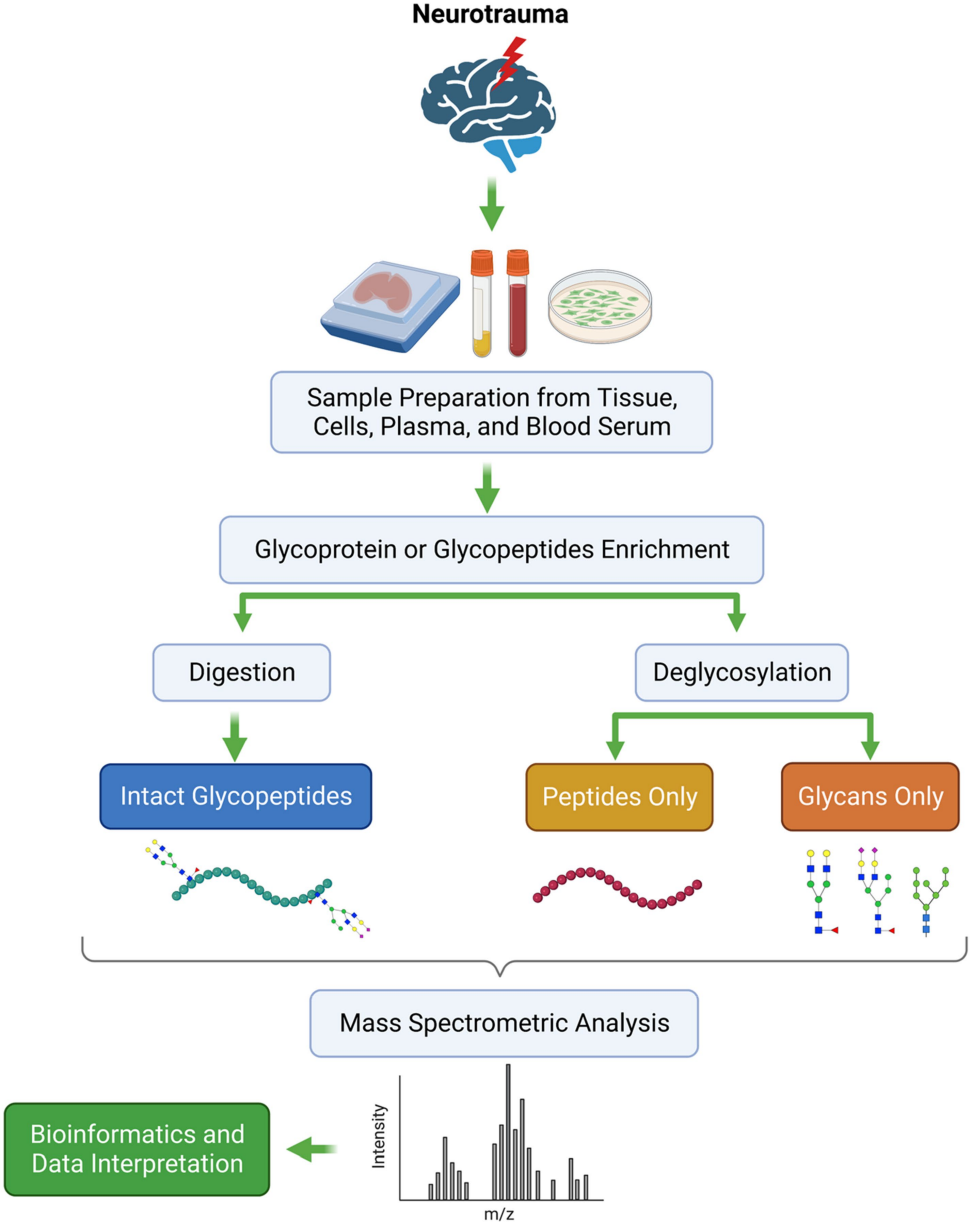
The general workflow of the MS-based glycoproteomics in neurotrauma samples.

It is pertinent to note that mass spectrometry provides valuable information regarding proteins with PTMs, and the importance of this is especially evident when comparing two or more samples quantitatively. Detecting changes in disease-associated glycosylation patterns requires sensitive, quick, reliable, and robust analytical techniques. Even though many methods are available for identifying glycoproteins, glycoproteomics remains a challenging field that holds much promise. With the advent of MS techniques, glycoprotein profiling has been significantly enhanced, especially when dealing with complex samples such as plasma, serum, and body fluids (77, 79). Recent advancements in MS technology have also made it possible to use more accurate approaches to characterize glycoproteins. A glycopeptide-based analysis provides site-specific information regarding the location of the glycan attachment on the protein, which is then used to determine its putative functional role and properties. Figure 3 illustrates the general workflow for analyzing glycoproteins in neurotrauma samples using separation techniques coupled with mass spectrometry.

### 1.3 Peptidomics

Peptidomics is a branch of proteomics that focuses on endogenous peptide fragments and is responsible for studying all peptides in a biological sample (80, 81). Peptidomics is considered a separate domain with its applications and analytic approaches (82). The term peptidomics was first used in February 2000 at the Association of Biomolecular Resource Facilities (ABRF) conference (83–85). The peptides’ life cycle is controlled by different processes and events, primarily determined by proteases and opposed-regulated protease inhibitors (85). Although peptidomics does not need enzymatic digestion in the preparation step, it is much more complicated than proteomics. A peptide in a peptidome analysis does not contain a uniform basic C-terminal (Lys/Arg) because of the absence of tryptic digestion used in proteomics approaches (86).

The ultimate aim is generally to classify all peptides, even though they originated from the same precursor (87, 88). Both fields, proteomics and peptidomics share the same limitation in dealing with the changeable features of the proteins and peptides. In a biological sample, peptidomics examination is usually hindered by protein degradation (enzymatically or non-enzymatically), interfering with the original peptides in that sample. Peptides are generally found at low levels, making any minor degradation of proteins interfere with the endogenous peptides’ signals in the MS analysis (89). To overcome this limitation, an affinity column for peptide enrichment can be used (90, 91). Moreover, heating tissues during extraction via an ordinary microwave oven result in the rapid inactivation of the proteases responsible for the protein degradation (92, 93). Tissues high in peptides and low in digestive enzymes, e.g., the pituitary, act as perfect targets (92, 93).

Neuropeptides affect several physiological functions, including body weight, sleep, anxiety, learning, and reward systems (94). Previously, neuropeptides were identified and quantified using several methods; one of these techniques was the radioimmunoassay (RIA) (95). It is an antibody-based sensitive technique that is not unique to a particular isoform of peptide. RIAs cannot distinguish between modifications if a specific antibody for the different isoforms is used (phosphorylation, sulfation, acetylation, and glycosylation) (96). The N-terminal sequences of proteins or peptides are determined using the Edman sequencing (97). The automated Edman degradation method was utilized in several different samples to sequence the neuropeptides (97). Although it was precise and its analysis was clear, it can only study pure peptides and remove peptides with N-terminal modifications (as a result of acetylation, formylation, or pyroglutamination, for example). Consequently, RIA has been entirely replaced by MS, which has higher throughput and sensitivity even with protein mixtures (98). MS usually use one of two standard techniques; the first one is electrospray ionization (ESI), which can produce multiple charge state for the separated ions, and the second one is MALDI, which lessen the complexity of identification because of single charged ion. Usually, peptides less than 10en kilodaltons (kDa) cannot be detected in a two-dimensional product (58, 60). polyacrylamide gel electrophoresis (2D-PAGE) that is used in the proteomics approach (99). High-performance liquid chromatography (HPLC) is a more reliable, precise, and easily automated technique that is considered adequate for separating peptides from mixtures (100). Micro or nanoscale LC avoids peptide co-elution in complicated mixtures and improves the low abundance LMW peptides identification (101).

Peptidomics reported significant progress, especially in neuropeptide studies, enabling the isolation and the classification of thousands of neuropeptides in one controlled experiment using LC–MS/MS. (102) Neuropeptidomics is a broad term for a method of characterizing neuropeptides on a global scale, often under particular physiological conditions. The first comprehensive analysis was achieved by Anna Secher et al. (103). Numerous (full-length) neuropeptides were analyzed by single LC–MS/MS analysis for 32 rats by perfusion protease inhibitors, which resulted in the identifications of 14,416 unique peptide sequences (104). The list of peptides was classified by their involvement in protein groups according to an established mammalian orthologous community structure to differentiate between neuropeptides and peptides produced from tissue damage (105). This allowed an overall grouping of high data levels from publicly accessible databases of proteins across different species (Uniprot.org, SwePep, and Neuropeptides.nl) (104). The initial peptide identification approach is based on data-dependent shotgun sequencing that compares the generated tandem mass spectra to an entire proteome database (103).

There are two types of peptidomics studies that can conclude the discovered peptides’ function. The first one is adhering to a singular cell or a specific cell type with a known function (such as pancreatic beta cells). The second one indicates the peptidomics quantifications (89). Neuropeptides levels, which can be affected by food intake, are directly correlated with physiological conditions. In fact, peptides can be considered as modulators for energy balance (89). MS can give the relative amount or intensity of each peptide either by isotope labeling and/or label-free quantification approaches (89), which will be discussed in the following sections.

Peptidome analysis was used to study and classify differentially expressed peptides in neonates with hypoxic–ischemic brain injury (HIBD) or controls in cerebrospinal fluid (CSF) in order to provide a basis for identifying new promising neonatal HIBD treatments (106). A total of 35 differentially expressed peptides were detected using (ITRAQ LC–MS/MS). A fragment of heat shock protein 90-alpha (HSP90α/HSP90AA1) has been shown to be a decreased peptide in neonatal HIBD (HSQFIGYPITLFVEKER) (106). This peptide, called Hypoxic–ischemic brain damage-associated peptide (HIBDAP), is a highly stable hydrophilic peptide in mammalian reticulocytes and has a 3.5-h half-life. This identification may significantly affect and contribute to the development of novel therapeutic targets for neonatal HIBD. Control CSF samples were taken from comparable infants with no identified neurological condition (106).

### 1.4 Proteomics and peptidomics technologies in personalized medicine

The primary aim of personalized medicine is to define diseases more accurately to support precise diagnosis and individualized medical treatments tailored to the patient characteristics and needs, ultimately maximizing efficiency and benefits (48). Proteomics-based personalized medicine is more complicated than genetic medicine and other omics fields because of the proteome’s complex nature and its dynamic components, PTM, tissue, cellular, and organelle-specific expression. Besides, the proteome profile differs in a healthy state compared to a disease state, especially in neurodegenerative diseases (107, 108).

Biomarkers are crucial to the advancement of personalized medicine. Since they may be used as a starting point for drug development and diagnosis, protein and peptide biomarkers may be used for early diagnosis and prevention strategies (109). They can also be used to monitor a patient’s reaction to therapy. Proteomics and peptidomics technology advancements may have aided the improvement of personalized medicine by recognizing protein and peptide biomarkers and improving biochemical diagnostics. The connection between diagnosis and treatment is critical for personalized medicine; proteomics and peptidomics are expected to show high efficiency in linking diagnostics and therapeutics (110). Further research is needed in this field.

### 1.5 Single-cell proteomics

Single-cell proteomics (SCP) is a technique used to understand the phenotype of a specific cell, which is a result of genetic interactions within this cell. Such a technique enabled the identification of a vast amount of proteins being expressed in a single cell at a certain point in time (111). Protein analysis of a single cell may show the presence or the progression of a disease through the detection of protein concentration in the blood or the detection of PTMs of a specific protein. Also, the change in the structure or the kinetics of a protein could lead to the knowledge of the disease progression, change in the immune response, and cell differentiation (112–114). Cellular heterogeneity denotes the protein expression in a disease or healthy state in which these proteins can be encountered as parameters in identifying the pathology of the disease (115). There are many methods used to detect proteins within a cell “proteome,” such as chromatography (e.g., liquid chromatography), gel electrophoresis (e.g., SDS-PAGE), and MS-based methods. Although these methods are potent, they are incompatible with single-cell detections, so it is unhelpful in the cell heterogeneity (1, 116).

SCP is a technology that identifies the complete protein profile within a single cell, the target cell, at a specific condition or time (111, 117). The benefits of single-cell proteomics rely on expanding our knowledge for understanding cellular functions and regulation with the characterization of protein phenotypes within the cell. A single-cell analysis requires sensitive methods and an optimized workflow starting with efficient sample isolation, liquid-phase separation followed by ionization, gas-phase separation, and MS. (118)

**Figure 4:**
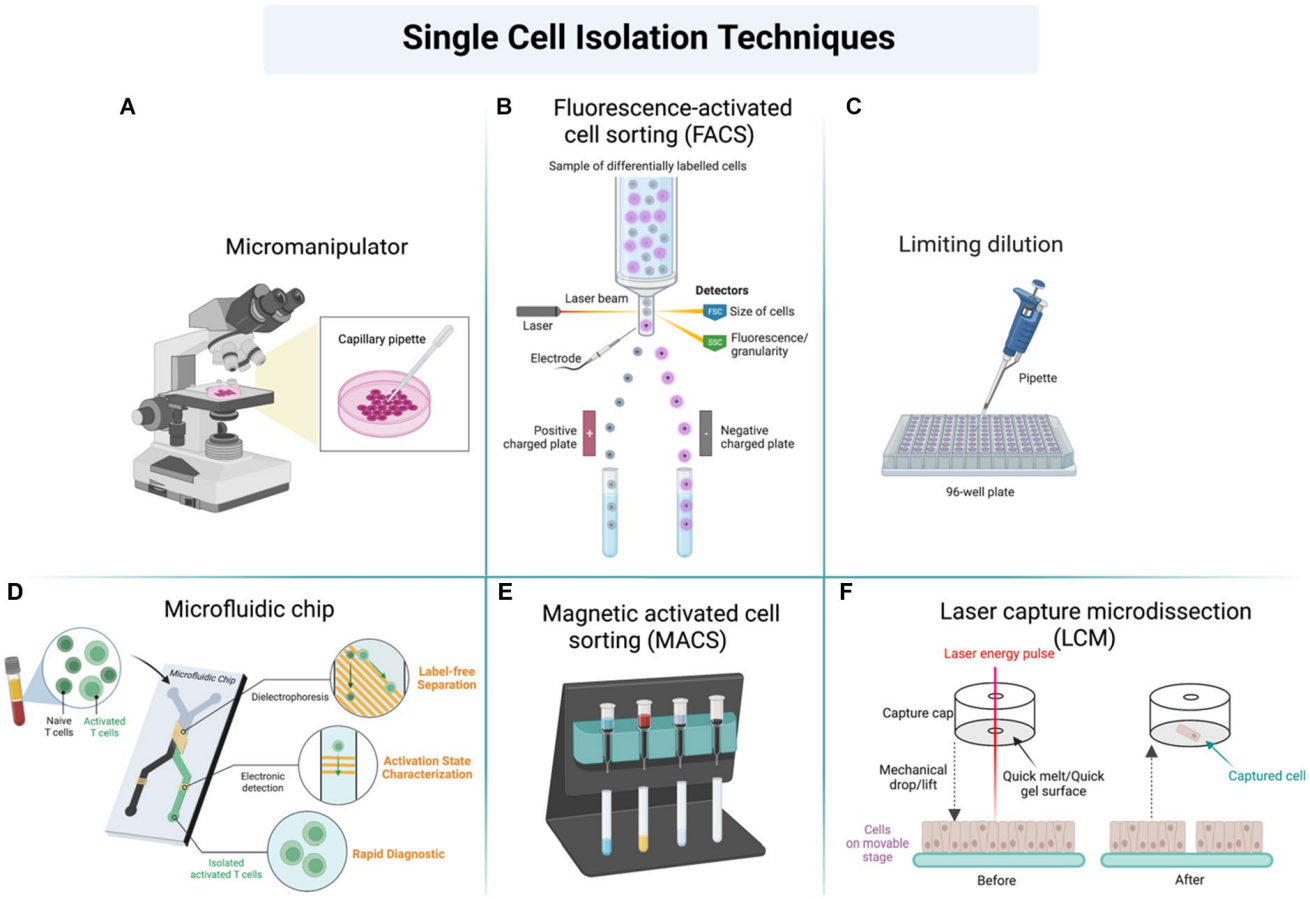
Different techniques used in sample preparation and cell isolation for further analysis of single-cell proteomics (SCP) by using **(A)** Micromanipulator, **(B)** Fluorescence-activated cell sorting (FACS), **(C)** Limiting dilution, **(D)** Microfluidic chip, **(E)** Magnetic activated cell sorting, **(F)** Laser capture microdissection (LCM).

To perform SCP, the single cell must be first isolated from a population of cells. The properties of the isolated cell must be established, such as cell size, cell density, and antigen status (111, 117). Techniques used in cell isolation for SCP are fluorescence-activated cell sorter, microfluidics, limiting dilution, manual cell picking, high-density microarray, laser capture microdissection, aptamer binding, and central magnetic-activated cell sorting (MACS) (113, 119) (Figure 4). Many reagents are used in the sample preparation for SCP, such as dithiothreitol (DTT), iodoacetamide (IAA), trypsin, and urea.

The separation of proteins is a necessary step in SCP, and it can be performed either using gel chromatography or electrophoresis. These conventional methods are impractical for the detection of low-abundant proteins. Other methods, such as capillary electrophoresis (CE) or microfluidic chips, are more precise in separating the whole proteome in a single cell (120).

The SCP’s analytical tools can be divided into either qualitative or semi-qualitative. An example of the qualitative method is the ELISpot technique, an antibody-based method where the antibody–antigen interaction detects the secreted proteins (121). The other semi-qualitative tool includes the flow cytometry methods (imaging and mass-based techniques) that detect many proteins expressed in a single cell (122–124). Techniques in the label-free analysis of SCP include matrix-assisted laser desorption/ionization (MALDI-MS), electrospray ionization MS (ESI-MS), secondary ion MS (SIMS), and laser/desorption/ionization (LDI-MS) (125, 126).

There are two alternative methods in sample preparation for the LC–MS label-free quantification of a single cell. These preparative methods, called integrated proteome analysis devices (iPad), inject the cell into the capillary (lysed and digested) that is coupled to nano LC–MS/MS. (127) It is very accurate in determining cell heterogeneity (128). The other method for the SCP sample preparation is using a pre-treated chip called the nanoliter-scale oil-air droplet (OAD). The OAD is based on microfluidics combined with proteomics shotgun analysis (129).

SCP in neurology was performed to assess microglial activation within TBI-induced animal models. Single-cell quantitative measurements assessed the activated and inactivated microglia. The morphology was changed in neurons following TBI insult, and the unaffected neurons had normal microglial density and morphology (130).

### 1.6 Quantitative proteomics

Quantification methods in proteomics include label-based quantification, such as the stable isotope labeling by amino acids in the cell culture (SILAC), tandem mass tag (TMT), iTRAQ, and the isotope-coded affinity tag (ICAT). Label-free mass spectrometric methods are also considered a quantification method (131, 132).

Stable isotopic labeling is one method in proteomics quantitation. It is based on applying a label to the peptides. The difference between labeled and unlabeled is mass, so the labeling can either be introduced in the culture (SILAC) or into fragmented peptides, called chemical labeling (133). The first labeling was introduced into Drosophila and *Caenorhabditis elegans* in the proteomics field by feeding (134). The SILAC quantitation method is combined with mass spectrometry and bioinformatics. It is well suited to be applied as a biochemistry-based approach (135). The principle of the SILAC is that there are two populations of cells. The first population contains light amino acids (light isotopes), and the other culture contains heavy amino acids (heavy isotopes of nitrogen, oxygen, and carbon). The heavy amino acids of the same peptides have the same properties except for the mass, so the two sets’ proteomes can be distinguishable by the mass shift (135). Many studies were performed to quantify proteins within the brain, such as a study that used SILAC labeling of Neuro2A cells (136). Another study quantified the phosphotyrosine associated with the neurotrophic factor (BDNF) by the concept of SILAC (137). SILAC labeling was also applied in TBI quantification, such as in a study by Wu et al. which aimed to detect and quantify the overall proteome within TBI-induced rat models. ^18^O-water labeling and mass spectrometry, the study could identify 1,002 common proteins (in control and TBI samples). These proteins are essential in cellular assembly and morphology. The study pointed out that 200 proteins were dysregulated in TBI samples, of which 124 were increased (up-regulated) and 76 were decreased (down-regulated). Up-regulated proteins were involved in the actin-related cytoskeleton and neurons’ structure, development, and transport. Down-regulated proteins were identified as enzymes associated with the glycolytic pathway, Kreb cycle, and oxidation phosphorylation (138).

Chemical labeling, such as iTRAQ, ICAT, and TMT, is another type of quantitation proteomics, called isobaric mass tagging, in which these added mass tags are labeled and detected in case of fragmentation by trypsin (139, 140). The advantage of labeling a peptide (by iTRAQ, ICAT, or TMT) is that this labeled peptide is detected in the mass spectrometry as a single peak, even when one or more samples are mixed (133, 141). iTRAQ proteomics was applied in many areas, including phospho-proteomics of plant cells (142, 143). The most significant advantage of iTRAQ labeling is that it can cover many peptides within the sample, affecting the sample and peptide identification and quantification of up to eight samples simultaneously. This labeling type also improved accuracy in detecting b and y ions in the MS/MS spectrum (132, 144, 145). Alternatively, the ICAT method, which is based on using isotope-coded affinity tags, has the same concept of labeling peptides, but what is unique is that it is specific for the enrichment of cysteine-containing peptides by comparing two samples with either heavy tag or light tag, these tags are labeled (146). The labeling of peptides using the ICAT method is performed at the sample preparation step before the digestion step, and it can be performed enzymatically (trypsin) or chemically. The labeled and digested proteins are then detected by mass spectrometry using peak intensity or peak area (146–148). Additionally, TMTs are also becoming more and more popular for large-scale proteomics studies. Such experiments, which focus on proteoform analysis in drug time courses or perturbation studies or in large patient cohorts, can greatly benefit from the reproducible quantification of single peptides across samples (149).

In TBI, assessing the protein changes in mild TBI patients was an essential step in qualifying and quantifying the proteins compared to a control. A study used chemical labeling (iTRAQ) to screen the global proteome within a rat’s brain. The animal model containing mild TBI (mTBI) showed that 237 proteins were changed significantly. Some of these proteins were associated with cAMP signaling (adenylyl cyclase pathway–a G protein-coupled receptor triggered signaling cascade used in cell communication), and some were associated with cell adhesion, autophagy, myelination, microtubule depolymerization, and brain development (150). In another study, TMT-based proteomics was utilized to screen the potential biomarkers of acute-phase TBI in rats (151). Based on proteomics findings, the acute phase of TBI showed significant influences on oxygen transport, acute-phase response, and negative regulation of endopeptidase activity. Additionally, pathways related to the scavenging of heme from plasma, binding, and uptake of ligands by scavenger receptors were highly enriched in all time points of TBI samples.

**Figure 5:**
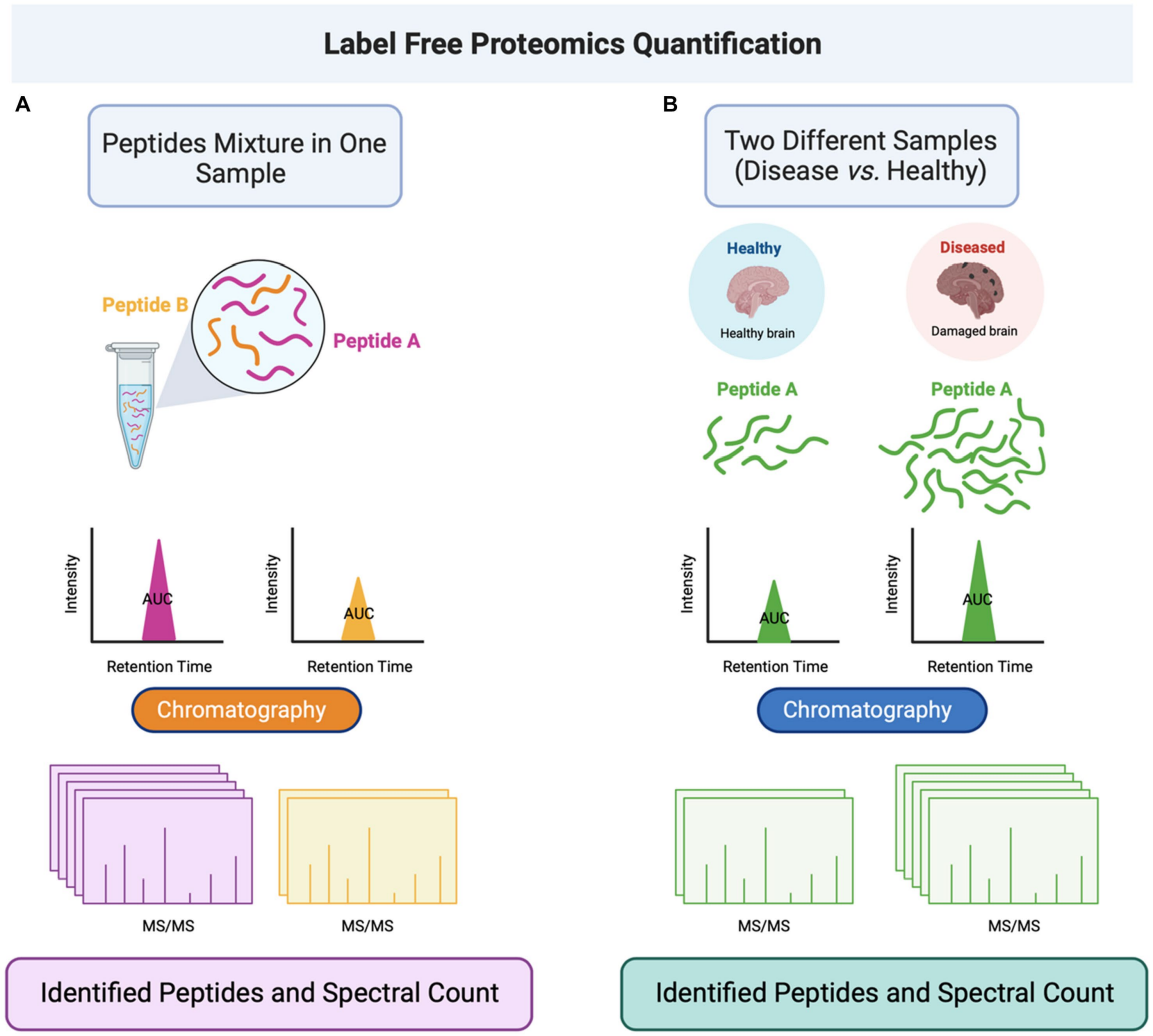
Label-free quantitation proteomics is performed by determining the area under the curve (AUC) of peptides eluted from the liquid chromatography and conjugated to MS/MS. **(A)** The label-free method can be applied to a single sample containing a mixture of peptides. **(B)** The method can also be applied to different samples (such as healthy and disease-representing cells) to quantify a specific peptide in the two cell populations. Abbreviations: Tandem mass spectrometry (MS/MS).

The label-free approach is another protein quantifying approach without labeling the peptides, which applies to all biological samples (152). This method needs long computational methods for independent mass spectrometry runs, which is required for data collection and interpretation (153). When label-free quantification methods quantify a mixture of peptides, the detection method can be performed by determining the area under the curve (AUC) or spectral counting (154). The AUC method is based on measuring the peak of an eluted peptide or protein from the chromatography (based on the retention time) (155, 156) (Figure 5). This method’s disadvantage is seen when there are multiple peptide signals. Also, this quantification method seems unhelpful with inaccuracy in the retention time within a specific peptide or the MS spectrum intensity. Also, some non-specific background noise affects the accuracy of the label-free methods (154). The second type, spectral counting, is based on selecting the most abundant peptides available within the sample for further fragmentation and generating spectra. The abundance of the peptide reflects the number of spectra it generates (157). The protein abundance index (PAI) factor calculation estimates the protein abundance, representing the number of peptides divided by the tryptic peptides of such a protein (158). A study developed the proteome platform for post-TBI neurons. The study aimed to report a quantitative assessment platform for the identified peptides using the label-free data-independent acquisition (DIA) method. The quantitation of 18,651 peptides revealed that 3,587 were statistically dysregulated upon TBI induction, and 634 (approximately 18%) were modified by PTM, such as phosphorylation and acetylation methylation (159).

Untargeted DIA mass spectrometry was also utilized to study whether examining the trajectory of TBI-responsive peptides secreted into urine could produce a predictive model of functional recovery during TBI rehabilitation (160). The produced models demonstrated high sensitivity and specificity, reflecting neuroplasticity and diminished cell death and neuroinflammation. The models can inform on rehabilitation progress after TBI and warrant further investigation. Besides, the acquired DIA mass spectrometry data used in a study on rats with mTBI showed that repeated injuries caused immediate cognitive problems, long-term movement issues, elevated levels of neurofilament light, changes to proteins in the hippocampus leading to brain inflammation, and widespread changes to white matter (WM) (161). Additionally, the other study aimed to investigate the differential effects of TBI if only the gray matter (GM) is damaged or if the injury also involves the WM. The researchers performed stab wound injuries affecting GM and WM (GM+) and one restricted to the GM (GM-) in the adult murine cerebral cortex and examined glial reactivity in the regions affected. Unbiased proteomics analyzes further corroborate their findings in support of a profound difference in GM reactivity when WM is also injured and revealed MIF as a key regulator of NG2 glia proliferation (162). Another study investigated the changes in axons during brain development in young rats and post-TBI in adult rats (163). The study found multiple similarities in the changes in axonal microtubule (MT) through tubulin post-translational modifications and MT-associated proteins (MAPs), such as tau and MAP6, during both development and TBI. Quantitative proteomics in this study uncovered similar signaling pathways of axon degeneration and growth/repair, including protein clusters and networks (163). This comparison approach shows how a focused examination of developmental processes can provide insight into the pathways initiated by TBI.

It is worth mentioning that neuroproteomics offers a global molecular approach to deducing the complex post-translational processes that underlie secondary events after TBI. By employing the DIA approach, there is a study that assessed the use of artificial neural networks and functional enrichment analyzes to discretize the temporal response across some 2047 significantly impacted proteins and supports the therapeutic promise of KCC2-targeted intervention for positive functional recovery after brain injury (164). Synthetic peptides are also used in quantitation, representing the same amino acid sequence of the desired peptide to be quantified. Stable isotopes can label these synthetic peptides upon proteolytic cleavage, and the labeled isotopic synthetic peptide will represent the amount of peptide within the sample. The advantage of this method is that it has a short analytical time with high throughput results (165, 166).

Many quantitative approaches are employed in proteomics to examine biological materials for relative quantitative proteomics, including label-free data-dependent acquisition (DDA) and isobaric multiplex labeling procedures (using iTRAQ or TMT reagents) (159, 167). Laser microdissection (LMD) or mechanical dissection can be used in neuroproteomics to extract brain areas for data collection from AD brains or control groups (168). DDA or isobaric multiplexed labeling techniques were used for sample preparation for relative quantitative proteomics and precise peptide measurements by LC–MS/MS, along with further data processing (165, 166). MaxQuant (169), Morpheus (170), and Perseus (171) are just a few of the database search engines available. DIA has taken the place of the DDA proteomics approach. For example, missing values can be solved using sequential window acquisition of all theoretical mass spectra (SWATH-MS). Implementing SWATH-MS quantification across the entire MS^1^ spectrum allows the classification of all measurable peptides within a given mass range. To eliminate missing values, the SWATH-MS approach enables an inclusive and reliable measurement of detected proteins in the sample (172–174). Therefore, MS^2^ spectra in DIA are more challenging to analyze than DDA spectra and need an advanced and specialized computation (174) to adopt this approach for quantification of complex brain proteomes in progress. This technique analyzes thousands of proteins in 1–2 h (175, 176). A recent study conducted a proteomics analysis on ventricular cerebrospinal fluid (vCSF) proteins following acute brain injury (ABI) and their association with pathophysiological pathways and potential biomarkers that can predict unfavorable outcomes (177). In this study, DIA and SWATH-MS was employed to compare differences in protein expression in patients with ABI and patients without ABI and in patients with traumatic and nontraumatic ABI. The results revealed that an unregulated expression of vCSF proteins after ABI could be linked to an increased risk of severe intracranial hypertension (ICH) and death. The study also identified specific vCSF proteins that were associated with increased inflammation, apoptosis, oxidative stress, and cellular response to hypoxia and injury.

### 1.7 Neuroproteomics in central nervous system injury

Different biomarkers correlate to different pathophysiological conditions. During neurological injuries, genetic modifications and ruptures of the blood–brain barrier (BBB) create post-translational covalent/ non-covalent protein modification in a variety of protein structures, such as glial fibrillary acidic protein (GFAP), myelin-oligodendrocyte glycoprotein (MOG), myelin-associated glycoprotein (MAG), ubiquitin carboxyl-terminal hydrolase isozyme L1 (UCH-L1), microtubule-associated proteins (MAP-2), neuron-specific enolase (NSE), α-II spectrin, and tau proteins. Different proteolytic fragments create endogenous high molecular weight (HMW) and low molecular weight (LMW) protein fragment mixtures within a variety of different biofluids (178). Endogenous proteolytic fragments can be detected in the nervous system cerebrospinal fluid (CSF), blood serum, saliva, urine, tears, and various other biofluids, all acting as potential samples for proteomics and diagnostic analysis (179).

### 1.8 Traumatic brain injury

Traumatic brain injury (TBI) is a major cause of health complications and death in young people and has a significant socioeconomic impact. When a severe TBI happens, there is a significant association with 30% mortality and disability among survivors (180). The severity of the TBI is categorized as mild, moderate, or severe, depending on many factors, such as hypoxic and ischemic damage, raised intracranial pressure, cerebral edema, infection, and hydrocephalus (181, 182). TBI’s signs and symptoms may include loss of consciousness, amnesia, nausea, dizziness, headaches, cognitive decline, structural brain damage, and other neurological symptoms (181). A brain injury, such as TBI, starts with applying mechanical force to the head, which can occur with or without loss of consciousness. This mechanical force then triggers a series of cerebral events that depend on the nature and location of the injury (183). The TBI mechanism starts with the impact on the brain tissue caused by mechanical force, which leads to the loss of cerebral vascular autoregulation that leads to abnormality in cerebral blood flow and metabolism. It also affects the mitochondrial function, which causes the accumulation of lactate and disturbs the balance of Ca^2+^, which affects the cell’s ability to maintain ATP (184, 185).

Neuroproteomics has also been efficient in investigating processes involved in CNS injury. TBI injury events occur in two phases mediated by different sets of proteins activating several pathways that shift the balance from pro-survival to pro-apoptotic/necrotic inflammatory states (186–188). These events are mediated by activated cysteine proteases that act on brain-specific proteins leading to an overall neural injury (189, 190). The neural cell death events involve primary and secondary injury phases involving different neural brain cells’ components accompanied by dysregulation of different neural proteins. The use of neuroproteomics applications on brain injury helps to understand altered protein dynamics, especially in biomarker research. TBI is a complex disorder that is hard to assess by current clinical techniques, including the computer tomography (CT) scan and magnetic resonance imaging (MRI), which are expensive instrumentations and are not universally available (191). Therefore, searching for brain injury biomarkers is crucial for diagnostic and prognostic purposes.

Many efforts were performed to clarify the complexity and progression of TBI. Sensitive and specific biomarkers are extensively used for the prognosis of neurotrauma. This can be achieved using immunoassays, including enzyme-linked immunoassay (ELISA) and western blotting (WB) (192).

Neuroproteomics, combined with bioinformatics, has shown to be a powerful tool in identifying pathways associated with TBI pathogenesis. Besides, it allowed scientists to figure out the biomarkers and drug target genes (2, 4, 5, 20, 193, 194). The identification and the analysis of blood biomarkers after TBI are maintained by sequential steps, starting with the release of proteins into the extracellular fluid or the blood from the damaged cells, where the detection of high-concentration biomarker proteins is measurable (21). Once the biomarkers reach the bloodstream, the exact concentration is determined by knowing the clearance rate or estimating the half-life of any protein.

In order to identify peptides within a cell, a separation technique must be applied, such as 2D-PAGE (194–198). An alternative method is the use of an ion-exchange chromatography (199). Mass spectrometry and chromatography showed more robust methods than conventional protein separation methods (200, 201). It was shown that mass spectrometry methods were more effective at tracing TBI-associated proteins to the disease progression, which would also make it easier to manage and target therapy for the disease (4). This powerful method separates the peptides according to the *m/z* ratio. These peptides are then identified by aligning them into a database. The antibody-based methods are an alternative to the mass spectrometry methods, divided into antibody microarray and immune-blotting.

A study reported identifying the global proteome in the hippocampal tissue by using SDS-PAGE-Capillary LC–MS/MS. (21) The global proteome strategy to identify and sequence neural biomarkers employed the cation-anion exchange chromatography, followed by 1D gel electrophoresis before the LC–MS/MS of the tryptic digested peptides (bottom-up) (11, 202). Proteome identification using the bottom-up method showed 59 proteins, which were differentially expressed. The study reported that 21 proteins were downregulated and 38 were upregulated. The global proteome strategy to identify and sequence neural biomarkers employed the cation-anion exchange chromatography, followed by 1D gel electrophoresis before the LC–MS/MS of the tryptic digested peptides (bottom-up) (20). For example, a proteome study identified 59 expressed proteins using the bottom-up method, which were differentially expressed and suggested that 21 proteins were decreased and 38 were increased (20). The MS/MS method showed high importance in identifying the change in protein expression and TBI progression (21).

Furthermore, quantitative proteomics was applied in a study to quantify the protein levels associated with TBI-induced cells, followed by targeted temperature management (TTM, mild hypothermia, 32°C). The study showed that by the label-based quantitation by iTRAQ, the proteins significantly associated with TTM were plasminogen, antithrombin III, and fibrinogen gamma chain transthyretin (203).

A study performed by Xu et al. was able to identify 4,031 proteins in TBI patients that are important in glial cell differentiation (e.g., myelin proteolipid protein and myelin basic protein), complement activation (e.g., complement decay-accelerating factor and complement C4-B), and apolipoprotein catalysis (APO) in the statin pathway (204).

Proteomics can also be used to identify therapeutic agents in TBI. One of the therapeutic agents is a Chinese medication called the XFZYD. LC–MS/MS, WB, and TMT-quantitation have been used to explore the mechanism of how the XFZYD is used to treat TBI and which proteins are targeted by XFZYD medication. The same study demonstrated by using bioinformatics before proteomics that “XFZYD” target proteins mainly involved in biological processes, cellular components, and molecular function (205).

In one study using 2D-PAGE coupled with matrix-assisted laser desorption ionization time-of-flight (MALDI-TOF) MS analysis, Siman et al. performed neuroproteomics analysis of CSF from the rat model with mild/moderate TBI (206). The results showed different proteins leaked into the CSF, including tau protein fragment of 17 kDa and αII-spectrin breakdown products (BDP150 and SBDP120). Another study by Burgess et al. located 229 proteins, 172 of which were novel hits, in healthy human postmortem immunoaffinity-depleted CSF (207). Results were validated by immunoblotting and sandwich enzyme-linked immunosorbent assay (ELISA) methods. Similarly, Kobeissy et al. designed an offline multidimensional separation platform termed cation-anion exchange chromatography followed by a 1D-PAGE separation (CAX-PAGE) (20). CAX-PAGE allowed for sample analysis without mixing to increase the mass range. This technique was tested on rat cortical samples and yielded results of 59 protein alterations and other novel protein breakdown products.

To investigate molecular pathological pathways underlying the progression of brain injury mechanisms, Yu et al. used a bioinformatics systems biology strategy based on assessing four distinct high-throughput gene expression studies of experimental TBI (208). Canonical pathways and the protein-interaction network were assessed as a scaffold to predict protein markers and identify novel molecular mechanisms involved in TBI. Results indicated that a subnetwork of 58 proteins related to synaptic capacity was identified, including postsynaptic density protein 95 (PSD 95), nitric oxide synthase 1 (NOS 1), and disrupted in schizophrenia 1 (DISC 1). These were validated using a penetrating ballistic-like brain injury rat model reaffirming the predictive bioinformatics model of protein interaction (208). In one study by Xu et al., where proteomics and bioinformatics techniques were combined, variations in protein expression levels were assessed in a Chinese TBI cohort (204). Tandem mass tags (TMT) labeling followed by LC–MS/MS was used to identify 4,031 proteins, including 160 that were overexpressed and 5 that were down-expressed. Upregulated proteins included myelin basic protein (MBP) and myelin proteolipid protein (MYPR), which play a role in glial cell differentiation pathways. Along with matrix metallopeptidase 9 (MMP9) and s100 calcium-binding protein A8 (S100A8) associated with inflammatory mechanisms.

Moreover, Thelin et al. evaluated the protein profile using an antibody bead suspension array in a rat model of severe TBI (209). During the initial day post-injury, complement factor 9 (C9) and complement factor B (CFB), which are involved in the innate complement system, were identified. Also, aldolase c (ALDOC) was found to be increased early on after the injury, while hypoxia-inducing factor (HIF)1α, amyloid precursor protein (APP), and Williams-Beuren syndrome chromosome region 17 (WBSCR17) protein were shown to be elevated weeks following the insult (209).

PTMs in TBI have also been assessed through proteomics, like in the case of one research by Lazarus et al., which focused on determining the brain regions susceptible to carbonylation (210). A study used the brains of female and male rats subject to injury through controlled cortical impact (CCI) and was immune-stained for protein-related structural changes. Directly in the injury site’s area, astrocytes and ependymal cells lining the third dorsal ventricle and the third ventricle floor above the median eminence displayed the highest protein carbonylation levels. The study presented male rats’ significant protein carbonylation at sites distant from the lesion, showing that hormonal protection is probable in oxidative stress. Ultimately, GFAP, dihydropyrimidinase-related protein 2, fructose-bisphosphate aldolase C (ALDOC), and fructose bisphosphate aldolase A (ALDOA) were identified in the study to be the most affected proteins by carbonylation in TBI. However, oligodendrocytes, microglia, and macrophages lacked this PTM (210). More recently, Mondello et al. have performed a comprehensive, in-depth profile and characterization of the *N*-glycome in serial blood samples of patients with moderate to severe TBI. This discovery study demonstrated a TBI-specific glycofingerprint reflecting molecular events and pathobiological mechanisms underpinning brain injury and recovery and contributing to patient endophenotyping. Moreover, *N*-glycans with important prognostic values that may represent novel targets for intervention were identified (57). This work paves the way for mapping the brain glycoproteome with the goal of enhancing our understanding of pathobiological mechanisms underpinning TBI and contributing to patient endophenotyping with significant implications for precision medicine.

### 1.9 Mass spectrometry imaging techniques

Neurotrauma, encompassing TBI and SCI, presents significant challenges in both diagnosis and treatment. Understanding the molecular changes occurring in the injured nervous system is crucial for developing effective therapeutic strategies. Mass spectrometry imaging (MSI) techniques, particularly MALDI, Desorption Electrospray Ionization (DESI), and SIMS have emerged as indispensable tools in neuroproteomics research, offering detailed insights into the molecular alterations following neurotrauma (211, 212). Moreover, the application of these techniques in precision medicine has paved the way for personalized interventions, optimizing treatment outcomes for individual patients (212, 213).

MALDI-MSI enables the comprehensive mapping of proteins, lipids, and metabolites in neurotrauma-affected tissues (214). In the context of TBI, MALDI-MSI has been instrumental in studying the spatiotemporal distribution of proteins associated with inflammation, neuronal damage, and repair processes (214). By analyzing specific protein expression patterns in different regions of the injured brain, researchers can identify potential therapeutic targets (215). Furthermore, MALDI-MSI facilitates the discovery of biomarkers indicative of injury severity and prognosis, aiding clinicians in making informed decisions about patient care and treatment strategies (212).

DESI-MSI offers distinct advantages in studying lipids, which play pivotal roles in neuronal membrane integrity, signaling, and inflammation (216). Following neurotrauma, lipidomic changes occur, influencing the progression of secondary injury processes (217). DESI-MSI allows for the direct analysis of lipid species in traumatized neural tissues, providing valuable information about lipid composition alterations (218). Understanding these changes is critical for developing interventions that promote neuronal survival and repair, making DESI-MSI a valuable tool in neurotrauma research.

SIMS imaging has also emerged as a powerful analytical technique in the field of neurotrauma research. By bombarding a sample surface with a focused primary ion beam, SIMS generates secondary ions representative of the sample’s elemental and molecular composition (219). In the context of neurotrauma, SIMS imaging offers unprecedented insights into the biochemical alterations occurring in injured neural tissues (216). Researchers utilize SIMS to map the distribution of specific biomolecules, such as neurotransmitters, lipids, and proteins, at subcellular resolutions (216). This detailed molecular profiling aids in understanding the complex mechanisms underlying neurotrauma, shedding light on cellular responses, metabolic changes, and signaling pathways associated with brain injuries.

In the realm of precision medicine, MSI techniques have far-reaching implications (220). By characterizing the molecular signatures of individual patients’ neurotrauma lesions, clinicians can tailor treatment approaches based on unique biochemical profiles. This personalized strategy enables targeted drug delivery, optimized rehabilitation protocols, and individualized neuroprotective interventions (221). Moreover, MSI can aid in monitoring treatment responses over time, allowing for adaptive modifications to therapeutic regimens and ensuring the best possible outcomes for patients suffering from neurotrauma (212). Therefore, these techniques have revolutionized neuroproteomics research in the context of neurotrauma. Their ability to unravel the complex molecular landscape of injured neural tissues not only enhances our understanding of injury mechanisms but also facilitates the development of personalized treatment strategies in precision medicine. By integrating these innovative techniques into clinical practice, healthcare providers can offer tailored interventions, ultimately improving the quality of life for patients affected by neurotrauma.

## 2 Technological advances in the field of neuroproteomics

In addition to the standard methodologies of biochemical and proteomics analyzes, technology and bioinformatics are rapidly being tied to medical research. There is a need for higher data analysis levels and evaluation, as the limitation of simple human-based trials and research is not sufficient for a comprehensive knowledge of the dynamic human brain. ML programs and AI present the opportunity to fill the gaps presented by traditional statistical analyzes, allowing for increased sophistication when dealing with complicated data sets. The field of computational analysis has only recently begun to gain traction within the biomarker research scope but shows clear promise in terms of application and utility.

ML is a term used to describe many computational models and AI programs that take user-defined information to generate predictions and other similar data (222). These models rely on a series of base algorithms that can quickly extrapolate results from datasets rich in complexity and volume. This makes them highly effective when dealing with topics that require a multivariate approach, such as clinical outcomes or novel data simulations. Additionally, many different ML analysis types allow a significant degree of freedom when addressing research direction. Many studies use several different ML algorithms, the main selections being: Random Forest, Decision Trees, Naïve Bayesian models, Logistic Regression (LR), Support Vector Machines (SVM), and Artificial Neural Networks (ANN). ANNs, LR, and SVMs are the programs that have been mainly used so far to explore outcomes within clinical research, each providing unique approaches to interpreting interrelated datasets (223). Most results show that the most effective analyzes are SVM and ANN, so these will be the main focus when discussing predictive ML models. LR will be discussed as it is the most common model that studies use as a comparison model because of its simplicity as a traditional ML program. LR is a widely used supervised learning tool that makes predictions based on input data’s logistic functions. However, ML techniques have recently outperformed traditional regression models when dealing with multiple datasets.

Chong et al. (224) ran a predictive analysis of 39 pediatric severe TBI cases. They aimed to develop a predictive model using a series of binary predictor variables, such as loss of consciousness and skull fracture. However, they ran two separate analyzes: one using traditional LR and another using a novel ML algorithm (225) designed initially to predict acute cardiac complications. They developed both models using parameters defined in their study and ran a ROC curve analysis comparing them. The effectiveness of an ML model can be measured by the relationship between its sensitivity (true positive occurrence) and specificity (true negative occurrence), as well as the AUC statistic generated by a ROC analysis of the generated results.

It was found that the ML model outperformed the LR model, most noticeable in terms of sensitivity (94.9% vs. 82.1%) and positive predictive value (90.2% vs. 72.7%). However, the ML model considered three extra variables that the LR model did not: clinical indication of seizures, confusion, and skull fracturing. When using an ML model, a risk to keep in mind is that the original predictions that ML algorithms learn from are derived from user-defined positive predictions. If the original predictions are flawed, the ML predictions may seem accurate but be just as flawed. These results are a testament to most ML models’ ability to handle large amounts of predictive data, more than most traditional statistical analyzes. Raj et al. (226) developed a pair of simple ML algorithms by expanding upon LR’s statistical concept. Instead of binary variables, clinical ICU measurements were used, namely intracranial pressure, mean arterial pressure, cerebral perfusion pressure, and Glasgow Coma Scale (GCS). One algorithm used the first three predictors, and the other included the GCS in its predictions. Designed as a prognostic tool for TBI patient mortality, these algorithms exhibited accuracies of 81% when identifying survivors and 84% when identifying deaths. Although this algorithm is simple compared to other ML models, it suggests that a move toward more dynamic and advanced analyzes could provide more effective tools to clinicians and researchers. Feng et al. (227) performed a more comprehensive comparative study in which 22 different ML models were compared to a LR model. The ML models included decision trees, discriminant analysis, SVM, and k-nearest neighbor algorithms. The goal was to predict the outcome of severe TBI patients using a combination of 40 different predictors defined as risk factors. When they performed their evaluations, the lowest accuracy rating for the ML models was 86.3% (quadratic discriminant), while the most accurate programs boasted an accuracy of 94% (linear, cubic, and quadratic SVM). LR showed an accuracy of 88%, falling short of 20 out of the 22 ML algorithms. ROC analyzes were performed, and LR showed an AUC of 0.83, while the average AUC of the ML programs was 0.82. However, when outlying algorithms that showed poor performance were removed (AUC 0.3, 0.47, 0.57), the average ML AUC was 0.88. The AUC values for the ML programs with the highest accuracies were 0.93, 0.94, and 0.93, respectively. This analysis supports ML algorithms’ use over LR models as prognostic tools in TBI. It also shows the enhanced performance of SVMs over the other ML models explored in the study. However, Gravesteijn et al. (228) found that there are no significant differences between flexible MLs and LR performance when dealing with a low number of predictors. They found that random forest models generate worse performances when compared to LR models under these conditions. They used IMPACT-II and CENTER TBI databases to perform their calculations, both of which have variables with thousands of data entries to use. Despite the large datasets, the low number of predictor values did not allow for increased performance, regardless of ML complexity. It is noted that in high-dimensional analyzes (using a high number of predictors), complex ML programs have been known to outperform LR. LR has still proven to be valuable for recent protein biomarker studies.

Thelin et al. (229) used a series of univariate LRs to compare the predictive usefulness of six protein biomarkers: S100B, NSE, GFAP, UCH-L1, tau, and neurofilament-light (NF-L). The programs’ predictions were dichotomized based on the Glasgow Outcome Scale (GOS) scoring (1–3 vs. 4–5, 1 vs. 2–5). UCH-L1 showed marginally better performance than the other biomarkers, with higher AUC values in all categories. The analyzes’ results were also used to assess trajectory curves and association strengths between each biomarker toward the outcome GOS scoring prediction. The biomarkers that were determined to have the highest predictive strength also showed the highest levels among patients with unfavorable outcomes, supporting the LR results. These biomarkers were also compared against traditional TBI predictors (GCS, CTC Scan, Glucose levels, etc.). Specifically, within the first 5 days, almost all biomarkers performed better as outcome predictors than the traditional criteria. Despite its simplicity and limitations, LR is still a handy prognostic tool. ML programs can create powerful new avenues for biomarker applications when coupled with temporal analyzes and the proper assessments.

Regarding dataset complexity, an element that is somewhat lacking among many TBI ML studies is continuous variables (Table 1). Much of the clinical data collected falls under binary or discrete variables, and the present continuous variables do not seem particularly specific to TBI prognostics. This disparity in data is partly because most traditional models cannot efficiently absorb large amounts of continuous data and compare it in a nonlinear or multivariate fashion. ML programs like SVM can become useful to remove this confusion in data organization. SVMs design a hyperplane based on the number of features or predictors used, and they then attempt to find a plane at the maximum distance between those points to develop a margin of error for future data points.

**Table 1:** Distribution of continuous and discrete variable use among ML studies.

Predictor variables	Outcome variables	Total	Continuous*	Discrete	Reference
Age, Motor GCS score, w, CT class, Traumatic subarachnoid hemorrhage, Epidural hematoma, Hypoxia, Hypotension, Glucose, Sodium, Hemoglobin	Death vs. Unfavorable Outcome (GCS < 4)	11	5	6	(228)
Age, Female, Primary mechanism of injury (Fall, Road traffic accident, Struck by projectile, Non-accidental injury), Loss of consciousness, LOC > 1 min, Difficult arousal, Vomiting, Seizure activity, Confusion/Disorientation, (Preverbal) irritability, (Verbal) Headache, (Verbal) Amnesia, Signs of altered mental status, Presence of unequal pupils, Clinical signs of skull fracture, Signs of base skull fracture, Presence of scalp hematoma, Frontal injury, Presence of scalp laceration, (Preverbal) with open fontanelle, Tense fontanelle	Predicted Risk Scores (0–100)	21	1	20	(224)
Mean arterial pressure, Cerebral perfusion pressure, Intercranial pressure, Glasgow coma scale	Death vs. Survival	4	4	0	(226)
Age, GCS (hospitalization), Injury severity score, Temperature, Systolic pressure, Diastolic pressure, Open brain injury or not, Concussion presence, Brain contusion presence, Brain-stem injury presence, Contrecoup presence, Epidural hematoma presence, Subdural hematoma presence, Hematoma volume, Intracerebral hematoma presence, Brain hernia presence, Oxygen saturation, Infection complication presence, Presence of other complications, Number of surgeries, Length of stay, Length of ICU stay, Multiple trauma presence, Tracheotomy presence, Period of mechanical ventilation, Aspiration presence, GCS (discharge), Previous TBI occurrence, Hypothermia presence, Acidosis presence, Presence of hospital-acquired pneumonia, White blood cell count, Dose of glucose, Glucocorticoid use, Nasogastric tube use, Coagulation change, Parenteral nutrition use, Lipid emulsion use, Enteral nutrition time, Sequelae or not	Death vs. Survival	40	10	30	(227)
Age, Sex, Helmet-wearing status, Coronary artery disease (CAD), Congestive heart failure (CHF), Cerebral vascular accident (CVA), Diabetes mellitus (DM), End-stage renal disease (ESRD), Hypertension (HTN) GCS score, Temperature, Systolic blood pressure (SBP), Heart rate (HR), Respiratory rate (RR)	Death vs. Survival	13	5	8	(230)
Age, Gender, Race, Mechanism of injury, Blood pressure, Heart rate, GCS on arrival to the emergency department (ED), CT scan findings, Injury severity score (ISS), The AIS per body region, Intubation status and location, Date/time of injury, Time of admission to the ED, Patients’ known comorbidities, Performed procedures, Blood transfusion, In-hospital complications, Outcome and date of disposition	Risk of Prolonged Medical Ventilation (PMV) PMV > 7 days vs. < 7 days PMV > 10 days vs. < 10 days PMV > 14 days vs. < 14 days	18	4	14	(223)
Subcortical, Auditory, Sensorimotor, Cerebellum, Visual, Salience, Executive control, Default mode network, Precuneus, language resting state networks	mTBI vs. No mTBI	10	0	10	(231)
Glucose, Hemoglobin, Albumin, C-reactive protein, Sodium, Urea, Magnesium, Lactate, Venous pH, White cell count (total), Neutrophil count, Hematocrit, Prothrombin time, Activated partial thromboplastin time	Favorable GOS (4–5) vs. Unfavorable GOS (1–3)	14	14	0	(232)
GCS, Systolic blood pressure (SBP), Abnormal pupillary response, Major extracranial injury, Cerebral contusion, Acute subdural hematoma (ASDH), Traumatic subarachnoid hemorrhage (TSAH), Epidural hematoma, and Skull fracture, Glucose, C-reactive protein, Fibrin/fibrinogen degradation products (FDP), Marshall CT classification	Poor Outcome vs. Good Outcome	13	4	9	(233)
GCS score, Systolic blood pressure, Heart rate, Respiratory rate, Temperature, Hematocrit, Age, Sex, Intubation status, ICD-9-CM injury E-code, and Injury severity score	Death vs. Survival	11	6	5	(234)
GCS score, Motor score, Eye-opening, Verbal response, Pupillary light reaction, Glucose level, Hemoglobin, Mass lesions, Cisterns, Midline shift >5 mm	Favorable (Alive with GOS > 3 at 6 months) vs. Unfavorable (Death or GOS < 3 at 6 months)	11	2	9	(235)
Neutrophil gelatinase-associated lipocalin (NGAL), N-terminal proB-type natriuretic peptide (NT-proBNP), Urine output (UOP), Plasma creatinine	Acute Kidney Injury vs. No Acute Kidney Injury	4	4	0	(236)
(After reduction) FA 2-OH C16:0, FA C18:0, TUDCA, PE ae C36:4, LysoPC a C20:4	mTBI vs. No mTBI	5	5	0	(237)
Age (< 65, 65) Gender, Histology (Adenocarcinoma, Squamous cell carcinoma, Large cell carcinoma, Undifferentiated carcinoma), T status, (T1, T2, T3), Regional lymph node status, (N0, N1, N2), Stage (I, II, III, IV), Grade (Low, Medium/high), Border of bronchus (Positive, Negative), FEV1 < 70%, Positive vessel infiltration, Positive lymphatic infiltration, Positive pleural infiltration, Chemotherapy adjuvant, Radiotherapy adjuvant, High expression of P53, High expression of caspase 3, High expression of-H2AX, High expression of Ki67	Death vs. Survival	32	0	32	(238)

*It should be noted that, unless otherwise specified, “Age” was treated as a continuous variable. However, it is hard to describe subjects’ age as being purely continuous because age is rarely reported as a continuous variable in most clinical settings. GCS, Glasgow Coma Score; CT, Computed Tomography; ICU, Intensive Care Unit; TBI, Traumatic Brain Injury.

SVMs are ML techniques that have begun to gain momentum recently. These programs excel at reading nonlinear relationships between input values if they are calibrated correctly. Kayhanian et al. (232) used SVM to design a 6-month prognostic tool for severe TBI in pediatric patients. They gathered patient blood test data and used 14 serum parameters as predictor values, all of which were non-discrete. The GOS was dichotomized (4–5 being good, 1–3 being poor) and used as the outcome variable. The maximal information coefficient and the absolute correlation coefficient for all parameters were found and plotted to narrow the algorithms’ focus. Three variables contributed noticeably more to the outcome predictions than the rest: glucose, lactate, and H^+^. Two SVM models were developed, one using all 14 parameters and the other using only the three highest contributors. The all-encompassing model exhibited a sensitivity of 63% and a specificity of 100%, while the triple-parameter model had values of 80 and 99%, respectively, making it the most accurate model. This study’s emphasis on measurable, continuous predictors in serum composition levels hints at the prospect of using SVM to predict outcomes and one for clinicians to use in mediation. Targeted interventions using these levels may help improve TBI outcomes rather than simply estimating them. Matsuo et al. (233) performed a similar analysis using a combination of discrete and continuous predictor variables. Fourteen different classifications were chosen, 3 of which fell under the category of regularly collected laboratory samples as in the Kayhanian et al. (232) study. These predictors were input into nine different ML algorithms, including SVM. The objective was to determine which ML program provided the best prediction for poor outcomes and in-hospital death and which predictors had the highest contributions to the models. This study’s results were varied compared to many of the other studies involving ML and TBI predictions. For the morbidity (poor outcome) model, random forest models showed the highest sensitivity (97.2%), and the highest specificity was achieved by the Gaussian Naïve Bayesian model (82.8%). The highest accuracy value was achieved by the Gradient Boosting Model (0.87). According to ROC analysis, SVM exhibited the third-highest sensitivity (0.97), the fourth-highest specificity (0.59), the second-highest accuracy (0.86), and the highest AUC (0.89). In the mortality models, SVM showed the second-highest sensitivity (0.78), the third-highest specificity (0.97), the highest accuracy (0.89), and the fourth-highest AUC (0.94).

No model proved superior when discerning the best ML algorithm, but the best predictors based on the models were age, GCS, fibrin/fibrinogen degradation product (FDP) levels, and glucose levels. Age and GCS are typically input as discrete variables, whereas the lab levels are continuous. This study shows that despite the diverse nature of the variables (discrete and continuous), the ML algorithms still showed good performances. The potential of SVM also reaches toward nontraditional means of prediction. An imaging biomarker study was conducted by Vergara et al. (231) compare resting-state functional network connectivity (rsFNC) to diffusion magnetic resonance imaging (dMRI). Brain scans of patients with mild TBI (mTBI) were used to test whether an SVM algorithm could be used to sort through image data and detect evidence of mTBI. Individual scans were defined to be separated into functional classifications (sensorimotor, visual, etc.) within the algorithm matrices. An SVM was then designed to separate the image data into two classifications: mTBI or healthy controls. The model that used rsFNC showed the most robust results, with an accuracy of 84.1%, a sensitivity of 89.4%, and a specificity of 78.8%. The models that used dMRI exhibited markedly lower performance. These results represent the potential for SVM beyond its functionality as a prediction tool. They show the ability to diagnose mTBI in a highly accurate manner via brain scanning and ML combinations, highlighting the importance of creative applications when using these programs.

While SVMs have proved to be powerful tools in many studies, other ML programs have proven superior under certain conditions. Rau et al. (230) performed a comparative study in 2018 to develop ML models that could predict mortality in isolated moderate to severe TBI (sTBI) patients. The study tested several different algorithms, including LR and SVM. However, this study also included ANN in its analysis. ANNs mimic the function of human neural networks to analyze large datasets. They are unidirectional and include inputs, at least one hidden layer, and output nodes.

Their complexity characterizes the major differences between ANNs and more in-depth deep learning; deep learning approaches have more hidden layers that are not immediately visible to researchers (labeled versus unlabeled datasets and algorithms). All the models tested in the study showed an accuracy above 90%, though the ANN showed a high value of 92%. The focus was shifted onto sensitivity, specificity, and AUC values because of the high accuracy ratings. The ANN’s sensitivity (84.38%) was about 20% higher than the second most sensitive algorithm. It also exhibited a high specificity at 92.8%, and its AUC was significantly higher than all other ML models at 0.97. For comparison, corresponding SVM values were 92.5, 65.6, 95.2%, and 0.93, respectively. Because of its high performance in both the training and the test sets across all measures, the ANN has been deemed the most effective prediction model. An ANN is more effective than typical injury scoring models such as TRISS when predicting survival for trauma patients (234) or GCS and GOS when giving 6-month TBI outcome predictions in children (235). The consistent strength of ANN when making outcome predictions in injury patients suggests its usefulness as a prognostic tool for clinicians, potentially more effective for clinicians and researchers to use over traditional injury scoring methods currently used. However, there are caveats. Raju et al. (239) stress the need for neurosurgeons to have competencies in neurosurgery expertise, statistical knowledge, and computation skills to utilize the potential of ML. Bertolaccini et al. (240) found that using ANNs in the medical literature has often been performed inaccurately, resulting in misleading results. ANNs do not require prior knowledge or statistical distribution assumptions to accurately establish input–output relationships (238), which acts as a great advantage with large datasets, especially nonlinear distributions. Compared to LR models, ANNs can have difficulty overfitting the model during the learning time and can be limited by computing power and available time when analyzing large datasets. Biomarker injury studies that use ANNs, especially within TBIs, are currently sparse.

However, Rashidi et al. (236) explored the possibility of using ML techniques as an early recognition system for acute kidney injury within burning and trauma patients. They combined it with exploring a novel polypeptide biomarker NGAL, along with other traditional blood AKI biomarkers (NT-proBNP, creatinine, and UOP). When testing the NGAL biomarker by itself, their ML methods proved to be extremely capable. Four of the five algorithms achieved an accuracy of at least 92%, with sensitivity values of at least 73% and specificities of 97%. The AUC for these models was at least 85%. They found that DNN (Deep Neural Network, an advanced type of artificial neural network) and LR models performed the best. Once a combination analysis of NGAL with other biomarkers was performed, performance statistics (particularly AUC) increased dramatically. DNN showed an incredible performance through its AUC values, with 7 out of 11 of the combined analyzes being at least 90%. The second cohort of patients was used to test for overfitting and other modeling errors. DNN performed noticeably worse with this cohort, with AUC values never reaching above 88% and having an all-time low of 49%. However, it was noted that this second cohort also contained non-burn trauma patients, which the DNN was originally not trained against. The introduction of novel characteristics would naturally hinder the trained DNN’s ability to make accurate predictions. The study speaks volumes about using ML programs, particularly neural networks when using blood and protein biomarkers. The generalizability of these programs to other sources of injury, such as TBI, marks the field’s potential (Table 2). Using several biomarkers at once in a highly efficient and automated analysis to output an equally accurate series of predictions paves the way for new avenues of prognosis.

**Table 2:** Summary of applied machine learning programs and corresponding receiver operating characteristic analyzes.

Machine Learning Tool	Number of patients	Application	Sensitivity	Specificity	AUC	G	Reference
LR based ML	472	Consecutive TBI in adults			0.810		(226)
					0.840		
LR	117	Severe TBI in adults			0.830		(227)
ML average (outliers removed)					0.880		
14 input SVM	94	Pediatric severe TBI	63%	100%	NA	37%	(232)
3 input SVM			80%	99%	NA	21%	
14 input LR			75%	99%	0.900	26%	
3 input LR			71%	99%	0.830	30%	
RF	232	Non-penetrating TBI with abnormal CT scan	97.20%	49.20%	0.860	53.60%	
Gaussian Naive Bayesian			68.70%	82.80%	0.842	48.50%	(233)
Gradient Boosting Model			93.70%	62.80%	0.857	43.50%	
SVM morbidity			94.50%	58.60%	0.894	46.90%	
SVM mortality			77.60%	97.00%	0.942	25.40%	
LR	325	Isolated moderate and severe TBI in adults	59.38%	93.54%	0.942	47.08%	(230)
SVM			65.63%	95.22%	0.935	39.15%	
DT			44%	98%	0.872	58%	
NB			59%	86.15%	0.908	54%	
ANN			84.38%	92.83%	0.968	22.79%	
RF	51	Acute kidney injury in burned and unburned adults	82%	68%	0.750	50%	(236)
k-NN			91%	82%	0.870	27%	
DNN & LR			91%	93%	0.920	16%	
LR	195	Moderate to severe pediatric TBI	82.10%	92.30%	0.930	25.60%	(224)
ANN			94.90%	97.40%	0.980	7.70%	
ESS	564	Chest pain patients	78.90%	76.50%	0.837	44.60%	(225)
DIST			63.20%	82.90%	0.720	53.90%	
MEWS			42.10%	78.50%	0.672	79.40%	
TIMI			78.90%	36.70%	0.621	84.40%	
LR	674	Prolonged mechanical ventilation following TBI	80%	68%	0.830	52%	PMV > 7 days (223)
SVM			83%	67%	0.800	50%	
RF			76%	70%	0.770	54%	
ANN			77%	60%	0.780	63%	
C.5 DT			70%	61%	0.650	69%	
LR			69%	79%	0.820	52%	
SVM	643	Prolonged mechanical ventilation following TBI	76%	82%	0.840	42%	Set B PMV > 10 days (223)
RF			81%	71%	0.800	48%	
ANN			76%	77%	0.770	47%	
C.5 DT			65%	75%	0.770	60%	
LR			29%	90%	0.750	81%	
SVM	622	Prolonged mechanical ventilation following TBI	29%	91%	0.740	80%	Set C PMV > 14 days (223)
RF			46%	80%	0.710	74%	
ANN			27.00%	94%	0.720	79.00%	
C.5 DT			25%	88%	0.650	87%	
rsFNC	100	Mild TBI imaging	89.40%	78.80%	0.841	31.80%	(231)
FA			76.60%	74.50%	0.755	48.90%	
FA + rsFNC			76.60%	72.30%	0.745	51.10%	
FA			70.20%	61.70%	0.660	68.10%	
			61.70%	66%	0.648	72.30%	
Marshall CT	565	Pediatric TBI CT scans			0.663		6-months unfavorable outcome (GOS < or = 3) (235)
Rotterdam CT					0.748		
Helsinki CT					0.717		
GCS Score					0.855		
Marshall CT	565	Pediatric TBI CT scans			0.781		6-month mortality (235)
Rotterdam CT					0.838		
Helsinki CT					0.814		
GCS score					0.920		
Linear SVM	632	Blood plasma predictors in mild TBI college athlete patients	81.70%	71.50%	0.830	46.80%	(237)
LASSO			77.80%	68.60%	0.811	53.60%	
MS/MS			69.50%	64.40%	0.738	66.10%	

ML, Machine Learning; LR, Logistic Regression, SVM, Support Vector Machine; ANN, Artificial Neural Network; DNN, Deep Neural Network; RF, Random Forest; (G)NB, (Gaussian) Naive Bayesian; GBM, Gradient Boosting Model; DT, Decision Tree; KNN, k-Nearest Neighbor; ESS, Ensemble-Based Scoring System; DIST, Euclidean Distance-Based Scoring System; MEWS, Modified Early Warning System; TIMI, Thrombolysis in Myocardial Infarction; GPLS, Generalized Partial Least Squares; RR, Ridge Regression; LASSO, Least Absolute Shrinkage and Selection Operator; MS/MS, Tandem Mass Spectrometry.

A key feature of ML programs is their flexibility of use. ML programs have proven to be very effective when making predictions using clinical datasets, but they also work well when combined with other advanced techniques, such as mass spectrometry. Mass spectrometry is a vital proteomics technique involving the fractioning of protein complexes via electrophoresis or chromatography. It allows for accumulating massive amounts of proteome data, making it particularly relevant to proteomics and genomics biomarker research (241). When coupled with powerful tools like ML, a wide variety of new explorative uses are made available. Fiandaca et al. (237) performed a study in which student-athletes were monitored for concussive events. Once an event occurred and a concussion was confirmed, blood plasma samples were taken less than 6 h after the event, 2 days after injury, 3 days, and 7 days. The samples’ metabolites were then isolated and identified using metabolic mass spectrometry. The metabolite readings were then analyzed using SVM, partial least-squares discriminant analysis (PLS-DA), and random forest analysis. The study aimed to develop ML programs that could isolate specific metabolites used as biomarkers for mTBI. The MS program originally came up with 2,811 possible metabolite biomarkers, which were reduced to 294 using MS reduction programs. This number was further reduced to the ten best-fitting metabolites using six different ML programs, including SVM and LR. These metabolites were analyzed among different athlete cohorts by the ML algorithms, and six main biomarkers were isolated as the best predictors of mTBI between all the study cohorts. These results represent a massive step toward rapid TBI diagnosis using a less invasive method (phlebotomy). This method also overrides the current diagnosis methods, such as GCS or GOS scoring, in terms of mechanistic application and quantifiable analysis. MS also has exhibited a remarkable ability for brain reconstruction. Nampei et al. (242) trained a series of ML programs to take in principle component analysis (PCA) data generated through MS and automatically reconstruct images of rat brains’ white and gray matter tracts. Mallah et al. (243) reconstructed and analyzed injured rat brains via MS to measure lipid differences within different post-TBI brain regions. A combination of these two studies’ techniques suggests the eve of a surge in biomarker research using MS and possibly ML to identify and spatially isolate potential biomarkers effectively. ML would also prove an effective tool in determining the biomarkers’ predictive and diagnostic value once data has been collected. It is essential to note in one area that current predictive clinical ML is found to be lacking in prediction “exactness. Many of the algorithms sort data into two categories: good or poor outcomes. It would be advantageous to design a classification system (possibly using GCS or GOS criteria) that ML could sort data into so that a particular value or prediction can be used to indicate certain symptomologies’ more detailed prognoses.

In addition, in the realm of neuroproteomics, AI has emerged as a groundbreaking tool, revolutionizing the way researchers analyze and interpret complex biological data (244). By harnessing the power of ML algorithms, AI enables scientists to process vast datasets with unparalleled efficiency and accuracy, leading to profound advancements in our understanding of the brain’s proteomics landscape (245, 246). One of the key applications of AI in neuroproteomics involves the identification and characterization of proteins associated with neurological disorders such as Alzheimer’s disease, Parkinson’s disease, and epilepsy (247, 248). Traditional methods for analyzing mass spectrometry data are often time-consuming and prone to errors. AI algorithms, however, excel at recognizing intricate patterns within these datasets, swiftly identifying potential biomarkers indicative of specific neurological conditions (10, 249). This capability not only accelerates the pace of biomarker discovery but also holds immense promise for early disease diagnosis and the development of targeted therapies, ultimately enhancing patient outcomes in the field of neurology (250).

Furthermore, AI-driven approaches in neuroproteomics extend beyond mere data analysis; they facilitate the prediction of protein–protein interactions (251), protein functions (252), and intricate signaling pathways within the brain (253). These predictions are invaluable for elucidating the underlying molecular mechanisms of neurological diseases. AI algorithms can integrate diverse omics data, including genomics, transcriptomics, and proteomics, enabling a comprehensive understanding of the complex interplay between genes, proteins, and pathways in neurological disorders (254, 255). Such holistic insights are pivotal for identifying novel therapeutic targets and designing personalized treatment strategies tailored to individual patient’s unique molecular profiles. As AI technologies continue to advance, their integration with neuroproteomics not only enhances our fundamental understanding of the brain’s intricate biology but also holds the potential to revolutionize clinical practice, ushering in a new era of precision medicine in neurology (256).

## 3 Neuroproteomics in personalized and precise medicine

With the development of benchwork proteomics studies and advancements in AI and ML, neurologists and biotechnologists are combining neuroproteomics and bioinformatics to develop the personalized medicine (226, 227). Personalized medicine expands precision medicine, creating unique prognostic, diagnostic, and therapeutic medical decisions customized uniquely for each patient (226, 227). With millions of patients every year, and individual responses from each patient, understanding how to respond to specific scenarios is complicated. Combining the use of biomarker quantitative statistical analysis, bioinformatics ML, and AI, the field of neuroproteomics can develop groundbreaking tools for diagnostic and theragnostic outputs for each unique individual (257, 258).

Today, physicians’ ability to diagnose specific diseases is limited, especially with complicated disorders such as TBI (mild vs. severe) (259). The genome variations, pathophysiological responses, and classifications for different people are currently unclear and undiagnosable. Most physicians utilize psycho-diagnostic scoring systems (such as the GCS and Marshall Scales) and compare their findings to those presented by neuroimaging (CT, MRI, and fMRI scans) (260). Limit ranges of knowledge and gray areas on the true difference between mild, moderate, and severe degeneration on a protein/psychological quantitative level are limited, leading to many false positive and false negative diagnoses (260). Utilizing hundreds of different variables and high-order machine organizations incorporates specific patients’ conditions, specialized gene variants, and various conditions on a personalized level. This organization and correlation schematic will help physicians understand how to proceed in certain neurological conditions (239).

**Figure 6:**
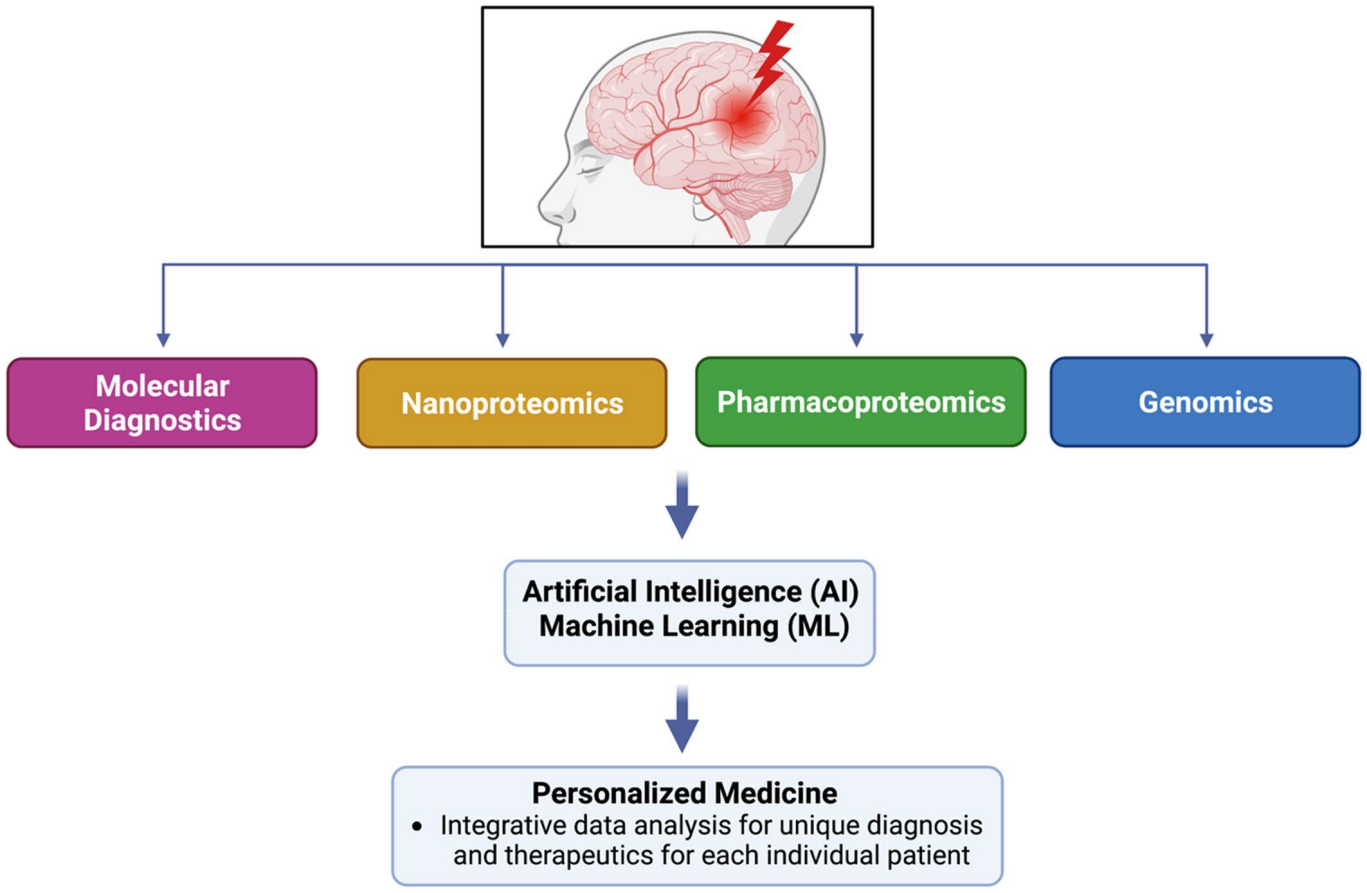
Artificial intelligence incorporated proteomics in personalized medicine. Once molecular diagnostic, nanoproteomics, pharmacoproteomic, and genomic data is collected correlations, AUC comparison, and significant tests are utilized to identify potential relationships between data. Data storage through AI and ML technology analyze large data sets to create faster and more accurate predictions with prognostic, diagnostic, and theragnostic value, all essential for effective personalized medicine.

Neuroproteomics incorporated personalized medicine can use AI and ML to analyze the interaction of hundreds of potential variables to provide the unique output for diagnosis and therapy. The AI and ML techniques described in the previous section can correlate specific protein concentrations, characteristic post-translational modifications, severity score index (SSI) ranges, injury types, time-dependence, protein-medicine interactions, and identify potential characteristics of diseases (259). Molecular diagnostic, nanoproteomics, pharmacoproteomics, genomics, metabolomics, and system biology data are all being collected in mass efforts for large databases (21). Taken together, the collection of mass neuroproteomics data and the development of higher-order systems will help classify precise neurological conditions for a more accurate form of personalized medicine (2, 20). Connecting the data collected from each field and associating patient characteristics through neuroproteomics technology will create more effective diagnostic claims for each unique patient, thereby advancing the field of personalized medicine (110, 257, 258) (Figure 6).

As precision medicine has been well adapted to genomics, proteomics precision medicine displays further complexity due to the proteome’s previously discussed dynamic nature. The steady control state’s proteome differs from the post-disease state that involved PTMs and protein–protein interactions. These intricate interactions are especially apparent in neuro-related disorders like AD and TBI. For example, AD displays a latent phase where no signs and symptoms are observed. However, pathophysiological processes still occur, leading to a prodromal phase with MCI and dementia (261). Identifying this latent phase marks a fundamental approach to dealing with the disease and requires tracking biomarkers to present critical steps in individualized medicine.

The application of neuroproteomics in clinical studies eliminates treatment response variability and provides a more targeted therapy approach. As the main goal of personalized medicine is to match the patient response with the best treatment to make sure the optimal outcomes are obtained, it is crucial to understand the challenges faced by proteomics compared to genomics, i.e., high cost and instrument robustness, to obtain the optimal outcomes (262). This is along with the fact that many biofluid proteomics studies rely on sample pooling, where result validation is performed by randomly selecting individual samples. As an example, the recent breakthrough in personalized medicine for AD as one of the significant public health issues involves the application of genomics and proteomics profiling to identify the specific biomarkers associated with the disease (263). Scientists have made significant progress in decoding the genetic variations and protein signatures unique to individuals with Alzheimer’s, enabling a more precise understanding of the disease’s heterogeneity (263). This personalized approach allows for early detection of Alzheimer’s risk, often before clinical symptoms appear, enabling timely interventions and lifestyle modifications. Moreover, targeted therapies tailored to the individual’s genetic and proteomics profile are being developed, aiming to slow down disease progression and improve cognitive functions. By customizing treatment strategies based on the patient’s molecular makeup, these advancements represent a paradigm shift in Alzheimer’s research and care, offering hope for more effective, personalized, and patient-specific interventions in the battle against this devastating neurodegenerative disorder (263).

In the realm of TBI research, precision medicine techniques have garnered substantial interest, although their translation into clinical practice remains a challenge (180). To date, only limited clinical studies have explored the potential of precision medicine approaches for TBI, and none have transitioned into the routine clinical application (264). Despite this, the scientific community is actively engaged in numerous preclinical studies, focusing on identifying and targeting the common genetic risk factors associated with TBI (264, 265). This formidable field offers several advantages translating into clinical research, a crucial step toward scientific applications (Table 3). Unlike the retrospective study of individual proteins/genes in post-mortem patient specimens, neuroproteomics allows for discovering novel proteins in a compassionate and unbiased manner. Remarkably, its ability to investigate these novel proteins does not require the formulation of specific hypotheses before experimentation. It also allows for building and analyzing extensive databases that allow data exploration from several centers. Hopefully, these ongoing investigations hold promise for the future, indicating a shift toward personalized therapeutic strategies tailored to individual genetic profiles, ultimately enhancing the prognosis and treatment outcomes for TBI patients.

**Table 3:** Pre-clinical utilization of neuroproteomics approaches in traumatic brain injury studies.

Animal subject	Injury type	Media tested	Techniques	Results	Validation	Reference
Sprague–Dawley rats	Fluid-percussion brain injury	CSF	2D-PAGE, HPLC, and MALDI-TOF/MS	60 proteins and their proteolytic fragments were identified to be released from cortical neurons associated with neurodegeneration. 14–3-3ξ, 14–3-3β, and 17-kDa fragments of tau were described as possible markers for acute brain injury.	Western blot analysis of conditioned media confirmed that 14–3-3ξ, 14–3-3β, CRMP-2, CRMP-4, and GAP43 were released in response to necrotic neurodegeneration.	(206)
Sprague–Dawley rats (*n* = 7)	Controlled cortical impact (CCI)	Brain tissue	Combined cation/anion exchange chromatography-PAGE, and reversed-phase LC–MS/MS	59 differential protein components were identified, 21 decreased, and 38 increased significantly in TBI. The former included collapsing response mediator protein 2 (CRMP-2), glyceraldehyde-3-phosphate dehydrogenase, microtubule-associated proteins MAP2A/2B, and hexokinase. Conversely, the latter included C-reactive protein, transferrin, and breakdown products of CRMP-2, synaptotagmin, and αII-spectrin.	Western blot analysis validated changes in protein levels and breakdown products.	(20)
Sprague–Dawley rats	CCI	Brain Tissue	Gel electrophoresis and MALDI-TOF/MS	Several mitochondrial proteins have undergone oxidative modification in the cortex and the hippocampus. In the cortex, the proteins are pyruvate dehydrogenase, voltage-dependent anion channel-2 (VDAC-2), fumarate hydratase 1, ATP synthase H+ transporting F1 alpha subunit. As for the hippocampus, the proteins are cytochrome C oxidase Va, isovaleryl coenzyme A dehydrogenase, enolase-1, and glyceraldehyde-3-phosphate dehydrogenase.	Western blotting validation was conducted for VDAC-2.	(266)
Sprague–Dawley rats (control/CCI male rats *n* = 7 and 9, respectively; control/CCI female rats n = 9 and 7, respectively)	CCI	Brain tissue	Fluid-phase isoelectric focusing, MALDI-TOF/MS, and immunohistochemistry	After injury, the injury lesion site, ventral portion of the dorsal third ventricle, and ventricular lining above the median eminence showed dramatic increases in protein carbonylation. The most susceptible for postinjury carbonylation were astrocytes and limited regions of ependymal cells adjacent to the dorsal third ventricle and the median eminence. Upon proteomics analysis, glial fibrillary acidic protein, dihydropyrimidinase-related protein 2, fructose-bisphosphate aldolase C, and fructose-bisphosphate aldolase A were the most affected by carbonylation in response to TBI.	N/A	(210)
Sprague Dawley rats (*n* = 73)	CCI After injury, the rats inhaled normoxic (22% O_2_) or Hypoxic (11% O_2_) air.	Serum	Targeted antibody suspension bead array	During the first days of injury, complement factor 9 (C9), complement factor B (CFB), and aldolase c (ALDOC) were detected at higher levels (*value of p* <0.01). In weeks period, hypoxia-inducing factor (HIF)1α, amyloid precursor protein (APP), and WBSCR17 were increased (*value of p* <0.05).	N/A	(209)
Sprague Dawley rats	Free-falling hammer (450 g) onto a stainless-steel disk attached to the rat’s head was used to induce injury	Cerebral brain tissue	iTRAQ and LC–MS/MS	1858 proteins were identified and quantified, where the comparative analysis identified 10 candidate proteins worth exploring. Out of these, citrate synthase, which binds to the inner membrane surface of the mitochondria and plays a critical role in the central metabolic pathway of aerobic organisms, was significantly downregulated 1 h post-injury. Similarly, synaptosomal-associated protein 25(Snap25) was downregulated 3d after the injury. Snap25 not only plays an important role in the regulation of synaptic vesicle exocytosis but it is also involved in axonal elongation.	Citrate synthase (CS), synaptosomal-associated protein 25 (Snap25), microtubule-associated protein 1B (MAP1B), and Rho-associated protein kinase 2 (Rock2), were validated by Western blot and immunohistochemistry analyzes.	(267)
Adult male Yucatan miniature and Yorkshire swines (*n* = 4 per group)	Blast tube was used to simulate free-field blast at a single moderate overpressure exposure of 40–52 psi	Frontal cortex brain tissue	LP-IEF, SDS-PAGE, and LC–MS/MS	Blast exposure was shown to affect deamination patterns in the brain proteome. 6 different proteins were shown to have undergone deamination, including GABA transaminase, aconitate hydratase, GFAP, Glutathione S-transferase, Histone H4, and vimentin. Additionally, the results showed a significant elevation of IgGs in the cerebral cortex.	Protein panels and IgG levels were validated by Western Blotting.	(268)
Male C57BL/6 J mice	Open-field low-intensity blast injury at a magnitude of 82 kPa	Brain tissue	TMT-tagging, LC–MS/MS	Mouse subjected to the blast displayed increased levels of tau protein phosphorylation 3 h to 24 h after injury. Analysis of phosphoproteins after the blast showed downregulation of 29 proteins and upregulation of only one protein at 3 h. However, four were downregulated, and 17 were upregulated at 24 h. The phosphoproteins with the largest increase include Add1, Camk2b, Syt1, and Stmn1. Ap3b2, Sgip1, Basp1, and Rph3a were among those significantly downregulated phosphoproteins.	N/A	(269)
Male Sprague Dawley rats	custom-designed device of metallic pendulum-striker was used to induce mTBI	Prefrontal cortex tissue	iTRAQ, HRPH, and LC–MS/MS	Analysis showed that 237 proteins were significantly changed in mTBI groups compared to the sham injury group. Significantly, two proteins, Pde10a and Gnal, which are involved in cAMP signal pathway, were suggested to play a role in mTBI pathogenesis. Both proteins were acutely upregulated after the injury and did not return to baseline levels until 6 months after the injury.	Western analysis of Pde10a and GnaI was done for validation.	(150)
Long-Evans rats	Repeated mild lateral fluid percussion	Plasma	Reverse Phase Protein Microarray	Plasma biomarkers indicated axonal damage, astroglia damage, oxidative stress, and metabolic dysfunction. There was a significant increase in GFAP (*p* = 0.029), 4-HNE (*p* = 0.003), NF-H (*p* = 0.004), and ceruloplasmin (*p* = 0.006) after injury. However, VEGF levels were found to be decreased (*p* = 0.002).	N/A	(270)
Male C57BL/6 J mice	Open-field low-intensity blast injury at a magnitude of 46.6 kPa	Brain tissue	TMT, LC–MS/MS	Results showed changes in 2216 and 459 phosphorylated proteins at various time points after the blast. Important pathways involved included evidence of mitochondrial dysfunction, oxidative stress, axonal/cytoskeletal/synaptic dysregulation, and neurodegeneration. Bioinformatic analysis identified blast-induced events related to cellular growth/development/movement/assembly and cell-to-cell signaling interactions. Notably, mitochondrial dysfunctions included impaired fission-fusion dynamics, diminished mitophagy, decreased oxidative phosphorylation, and compensated respiration-relevant enzyme activities.	Changes in mitochondrial markers associated with oxidative stress and fission-fusion dynamics at different time points post-injury were validated through western blotting.	(271)
Male Sprague–Dawley rats	Mild lateral fluid percussion injury (FPI), traumatic axonal injury (TAI)	Brain tissue	DIA LC–MS/MS	Results showed that developmental changes and TBI can cause modifications in axonal microtubules (MTs) through post-translational changes in tubulin and MT-associated proteins (MAPs) such as tau and MAP6. Degenerating axons show instability and depolymerization, while nearby axons without degenerating morphologies show enhanced MT stabilization. Further study is needed in this area.	Changes in tau and MAP6 expressions during development and after TBI, and also axon degeneration changes and tubulin PTMs were validated by western blotting, and IHC staining.	(163)
Male Long-Evans rats	Awake closed head injury (ACHI), repeated mild TBI	Tissue and Serum	DIA mass spectrometry, MRI	Repeated mTBI rats had acute cognitive deficits and prolonged sensorimotor impairments. Serum NfL was elevated at 7 days post-injury, correlating with sensorimotor deficits. Several hippocampal proteins were altered by repeated mTBI, including those associated with energy metabolism, neuroinflammation, and impaired neurogenic capacity. Diffusion MRI analysis at 3.5 months found widespread reductions in white matter integrity.	N/A	(161)
Adult male Sprague–Dawley rats	Mild–moderate controlled cortical impact (CCI)	Brain tissue	Ion mobility DIA mass spectrometry, Immunoblot analysis, Immunofluorescence microscopy	This study used artificial neural network and functional enrichment analyzes to identify ion transporters that could help address abnormal GABAergic transmission and delayed decline following brain injury. They focused on KCC2 or SLC12A5, and tested a KCC2-selective modulator CLP290. The modulator was effective at restoring lost KCC2 localization and improving somato-sensory behavioral tasks, but timing of administration was crucial. Results suggest the importance of post-translational characterization in developing TBI treatments, and the promise of KCC2-targeted CLP290 intervention for positive functional recovery after brain injury.	Validation of the escalated loss of KCC2 through the first week was confirmed through immunoblotting. However, the decrease in total protein was observed to be delayed until the second day, which was affirmed across two independent antibodies.	(164)
Adult C57BL/6 mice	Stab wound injury affecting gray matter (GM) and white matter (WM)	Brain tissue cerebral cortex	Confocal laser scanning, Isolation and fluorescence-activated cell sorting, DIA mass spectrometry	The study examined glial reactivity in regions affected by WM and GM injuries and compared the impact of WM injury on reactive gliosis in the GM. Results showed that microglia proliferation increased in the WM compared to GM in the GM+ injury, while proliferating astrocytes were more abundant in the GM. WM lesion strongly influenced the proliferation of GM glial cells, particularly at early stages post-lesion. The study also found that NG2 glia proliferation was decreased in the GM+ compared to the GM-lesion condition and that MIF regulates NG2 glia proliferation.	N/A	(162)

## 4 Neuroproteomics human clinical trials

Finally, in addition to neuroproteomics analysis abilities in labs and animal models, neuroproteomics has recently become a significant field in clinical practice. Many studies have used neuroproteomics for clinical diagnostic tools and theragnostic approaches. The use of human clinical research is key to seeing how biomarker detection within biofluids can be used for testing effective diagnosis, prospective therapies, and potential human cures. Clinical trials take the techniques and treatments developed in scientific bench work and apply them to animal and real-life human scenarios. Clinical trials require subject to consent, approval, and adequate resources. Once these are acquired, clinical trials are mandatory to develop potential cures and treatments, the next steps in the neuroproteomics research (2, 272, 273).

Neuroproteomics clinical trials have progressed over the last couple of years, with many testing novels and more efficient methods for diagnosing TBI and other neurodegenerative diseases. With hundreds of clinical trials introducing neuroproteomics approaches, the field is continuing to expand rapidly. Today, neuroscientists utilize neuroproteomics and protein correlation analysis to record protein levels and immunological responses to several disorders and symptoms within TBI and its relative proteinopathies (amyloidosis, prion diseases, tauopathies, Alzheimer’s disease, Parkinson’s disease). Multiple clinical trials have already evaluated variables such as demographic factors (pediatric TBI, geriatric TBI, age-related TBI, race, gender), injury types (concussion, mTBI, moderate TBI, sTBI), military TBI, blast wave TBI, injury severity diagnostic matrix scores including the GCS, Marshall CT scales, Pediatric Glasgow Scales, and Standardized Assessment of Concussion (SAC), vitals (temperature, weight, height, biomarkers concentrations, injury phenotypes), and a variety of different factors. AI and ML categorization of multiple variables and effective personalized medicine has led to effective prognosis, analysis, and diagnosis of different neurodegenerative diseases (2, 274–282).

Neuroproteomics and clinical research continue to utilize new biofluids, different biomarkers, and various tests to identify new correlations and algorithms for significant diagnostic measures in the development span. In recent human clinical trials, neuroscientists have taken amounts of blood, cerebrospinal fluid (CSF) samples, and other biofluids from patients in hopes of collecting and analyzing different biomarker levels (21, 283) (Table 4).

**Table 4:** Human clinical trial neuroproteomics.

# Of Subjects/patient demographics	Biofluid tested, proteins and clinical factors	Tests	Results/level of evidence	Data category	Institute	References
504 NCAA college athletes with concussions and control athletes (contact/non-contact sport athletes)	Blood GFAP, UCH-L1, and Tau levels recorded between 24 to 48 h after injury, and 7 days after return to play	Quanterix SIMOA multiplex assays, SCAT-3, SAC	Athletes with concussions had significant elevation in GFAP (*p* < 0.001), UCH-L1 (p < 0.001) and tau levels (*p* = 0.004). Supports the analysis of GFAP, UCH-L1, and Tau biomarkers as potential signals for concussion/ mTBI in contact sports.	Prognostic/Diagnostic Data (Concussion/mTBI)	NCAA and CARE	(278)
218 adult sTBI patients; (CSF from 138 subjects/Serum from 80 subjects)	CSF (EVD)/Serum temporal S100b profiles, recorded over 6 days post-injury. Evaluates correlations in, protein levels, age, gender, weight, & ISS.	GOS, DRS, ELISA	CSF and serum S100b levels were elevated over healthy controls across the first 6 days post-TBI (*p* ≤ 0.005 and *p* ≤ 0.031), all correlated with higher mortality and lower GCS scores. S100B levels provide significant predictions in sTBI cases.	Predictive and Prognostic Data (sTBI)	University of Pittsburgh Department of Physical Medicine and Rehabilitation	(276)
54 Geriatric Subjects (μ-age = 69–70); 23 with AACD, 16 with AD, and 15 healthy controls	CSF, Cerebral Gray Mater t-tau and p-tau181 concentrations correlated with AACD/ AD diagnosis, and MRI scans	ELISA, MRI, Voxel-based morphometry	AACD/AD subjects presented elevated t-tau and p-tau181 concentrations in respective areas of the brain. Study supports the elevated distribution of tau and p-tau in respective area of the brain for AACD and Alzheimer disease patients (*p* < 0.001).	Diagnostic Data (AD)	University of Heidelberg Geriatric Psychiatry and University of Frankfurt Department of Psychiatry	(280)
27 sTBI Pediatric subjects (ages 2–17 yrs.) with Glasgow Coma Scale score of ≤8	Serum/CSF GFAP levels correlated with Hypothermia treatments	GCS, GFAP quantification	CSF GFAP (15.5 +/− 6.1 ng/mL) and serum concentrations (0.6 +/− 0.2 ng/mL) were successfully utilized for predicting Pediatric Performance Category Score over CT images (*p* = 0.008). Elevated GFAP concentration patterns indicated prognostic data for sTBI. Control comparison of GFAP also indicated nonsignificant decreases in concentration using hypothermic interventions.	Prognostic &Treatment Intervention Data (sTBI/ pTBI)	University of Western Ontario Division of Critical Care Medicine, Department of Pediatrics	(275)
217 TBI patients (161 men/ 35 women); 196 of the patients were admitted with Level 3 acute TBI (< 24 h post-injury) while 21 patients were admitted to inpatient rehabilitation units (μ = 176.4 days post-injury).	Plasma p-tau/t-tau biomarker levels compared to CT scans and GCS scores	GSC and AUC	Total (T-tau) and P-tau levels differentiated mTBI from other forms of TBI. Illustrated that p-tau levels and the ratio of phosphorylation outperformed diagnosis from CT scans (AUC = 0.921 and 0.646, respectively). Proteomics supported p-tau as a stronger biomarker for acute TBI in comparison to t-tau or CT diagnosis.	Prognostic/Diagnostic Data (mTBI/ acute TBI)	State University of New York Laboratory of Neurodegenerative Diseases and CNS Biomarker Discovery (TRACK-TBI Pilot Study)	(279)
145 sTBI patients evaluated (< 6 h post injury). Serum samples from 86 patients and CSF samples from 59 patients	Serum/CSF UCH-L1 levels taken every 6 h.	ELISA	Supports a statistically significant increase of UCH-L1 levels overtime and respective biokinetics in CSF and serum samples for severe TBI patients in comparison to controls (*p* < 0.001), indicating a highly potential indicator of time dependent classification of sTBI.	Predictive data (sTBI)	University of Florida Center of Innovative Research, Banyan Biomarkers Inc., and Department of Psychiatry	(274)
28 pediatric patients	Serum S100b, NSE, IL-6, IL-8, IL-10, SICAM, L-selectin, and endothelin levels 1 Day post-injury	ELISA, GOS, ROC, Multi-ROC, and AUC,	(1) Combining biomarker levels for S100b (“screening marker”) and L-selectin/IL-6 (“varying markers”) achieved an AUC =0.98 when predicting outcome predictions. (2) Specificity and sensitivity for unfavorable outcome prediction were 96 and 100%, respectively. (3) Supports the use of multiple biomarkers (like S100b and IL-6) as potential prognostic tools for post-traumatic outcome projections.	Prognostic & predictive data (predicting unfavorable outcomes of pTBI)	University of Edinburgh Child Life and Health	(277)
217 TBI Patients 161 men; 35 women; 196 patients with acute TBI and 21 subjects with chronic TBI	Plasma AutoAb [GFAP] response levels in post-acute/chronic TBI.	Mini-Protean II Multiscreen and Immunoblot Screening	AutoAb [GFAP] levels for subjects with chronic TBI (176 days post-TBI) were significantly higher (15.08 ± 2.82; *n* = 21) than healthy controls and slightly higher than acute TBI patients (*p* < 0.001). Illustrates the significantly increased levels of autoimmune response and AutoAb [GFAP] levels in chronic TBI due to GFAP protein fragmentation.	Diagnostic Data (Acute and Chronic TBI)	University of Florida Departments of Psychiatry and Neuroscience (TRACK-TBI)	(282)
50 ABI patients (23 SAH, 15 TBI, 6 intracranial hemorrhage, 3 ischemic stroke, and 3 others) and 12 patients without ABI	Ventricular cerebrospinal fluid (vCSF), prospective study on the protein expression	DIA-SWATH mass spectrometry	The study found significant protein expression differences between patients with and without ABI. Patients with severe intracranial hypertension or death had higher GFAP expression than those without. Differences in protein expression were also found between patients with traumatic and nontraumatic ABI, with some proteins related to structural damage, complement activation, and cholesterol metabolism. However, no significant differences were found in protein expression between patients with SAH versus TBI or between those with good versus poor 3-month Glasgow Outcome Scale score.	Biomarker Discovery	Universite Libre de Bruxelles, Department of Intensive Care, Erasme Hospital	(177)
12 patients with moderate to severe TBI (initial Glasgow Coma Scale ≤12)	Urine, multivariate urinary peptidome	DIA mass spectrometry	Highly sensitive and specific models were created using top 20 discriminant peptides for DRS-and FIM-based models with area under the receiver operator curve of 0.99 and 0.95, respectively. Predictive ability was assessed using robust leave-one-out cross-validation with Q2 statistics of 0.64 (*p* = 0.00012) and 0.62 (*p* = 0.011) for DRS-and FIM-based models, respectively, both with a high predictive accuracy of 0.875. These models can help track rehabilitation progress after TBI and should be studied further for efficacy in assessing therapeutic interventions.	Biomarker Discovery, Predictive model of functional recovery during TBI rehabilitation	Virginia Commonwealth University, School of Medicine	(160)

AUC, Area Under the Curve; AACD, Aging-associated Cognitive Decline; AD, Alzheimer’s Disease; NCAA, American National Collegiate Athletic Association; CSF, Cerebrospinal Fluid; CT, Computed Tomography; DRS, Disability Rating Scale; ELISA, Enzyme-linked Immunosorbent Assay; EVD, External Ventricular Drain; GFAP, Glial Fibrillary Acidic Protein; GOS, Glasgow Outcome Score; GCS, Glasgow Coma Scale; ISS, Injury Severity Score; IL-6, Interleukin-6; IL-8, Interleukin-8; IL-10, Interleukin-10; MRI, Magnetic Resonance Imaging; mTBI, Mild TBI; NSE, Neuron-Specific Enolase; pTBI, Pediatric TBI; p-tau, Phosphorylated Tau; ROC, Receiver Operating Characteristic; S100b, S100 Calcium-binding Protein B; sTBI, Severe TBI; SICAM, Soluble Intracellular Adhesion Molecule; SCAT-3, Sports Concussion Assessment Test-3; SAC, Standardized Assessment of Concussion; t-tau, Total Tau; UCH-L1, Ubiquitin C-terminal Hydrolase-L1; CARE, US Department of Defense Concussion Assessment, Research, and Education Consortium.

It has been demonstrated in clinical trials that biofluids and protein biomarkers provide valuable information for pathophysiological prognostic, and diagnostic (Table 4). Compared to all the different types of biofluids extracted from clinical trial subjects, CSF serum seems to be the most useful in finding correlations between neuropathy and TBI, as it provides the most direct data from the CNS (283). Most trials utilize the cadaver CSF samples; however, recent studies have also utilized patients’ consent to take samples from external ventricular drains (EVD) (283). With EVD, patient CSF is drained from catheters as a routine procedure to reduce intracranial pressure (ICP). EVD and ICP reduction is systematic and causes no harm to the patient while also helping obtain potential samples for the clinical neuroproteomics research (276, 284, 285). Although less invasive, blood samples, urine, saliva, and many other biofluids are also collected to test respective proteins (276, 285). Each biofluid provides valuable concentrations of different biomarker proteins, necessary for proteomics analysis. Different biofluids and biomarkers have been collected and analyzed, leading to groundbreaking algorithms for physicians’ prognosis and diagnosis (272, 286) (Table 4).

Today, various peptidomics approaches have been investigated successfully, as discussed earlier. Scientists continue to find correlations between high levels of specific proteins and their links to potential diseases (286). Levels of specific biomarkers such as tau, p-tau, GFAP, UCH-L1, and many others have been critical for clinical predictions (25, 279, 287, 288). Many studies have already associated different biomarker trends with specific age groups (adult TBI, pediatric TBI, geriatric TBI), injury types (blast injury, military injury, physical injury, gunshot, sports), and times of recovery (1 h, 2 h, 8 h, 12 h, 24 h, 48 h) (274–280, 282). The use of proteomics, as seen in many correlational studies, is vital for the future prognostication and diagnosis of a variety of different types of currently undiagnosable TBI: mTBI, moderate TBI, sTBI, pediatric TBI, geriatric TBI, and a variety of other neurotrauma (274, 277, 280, 289).

**Figure 7:**
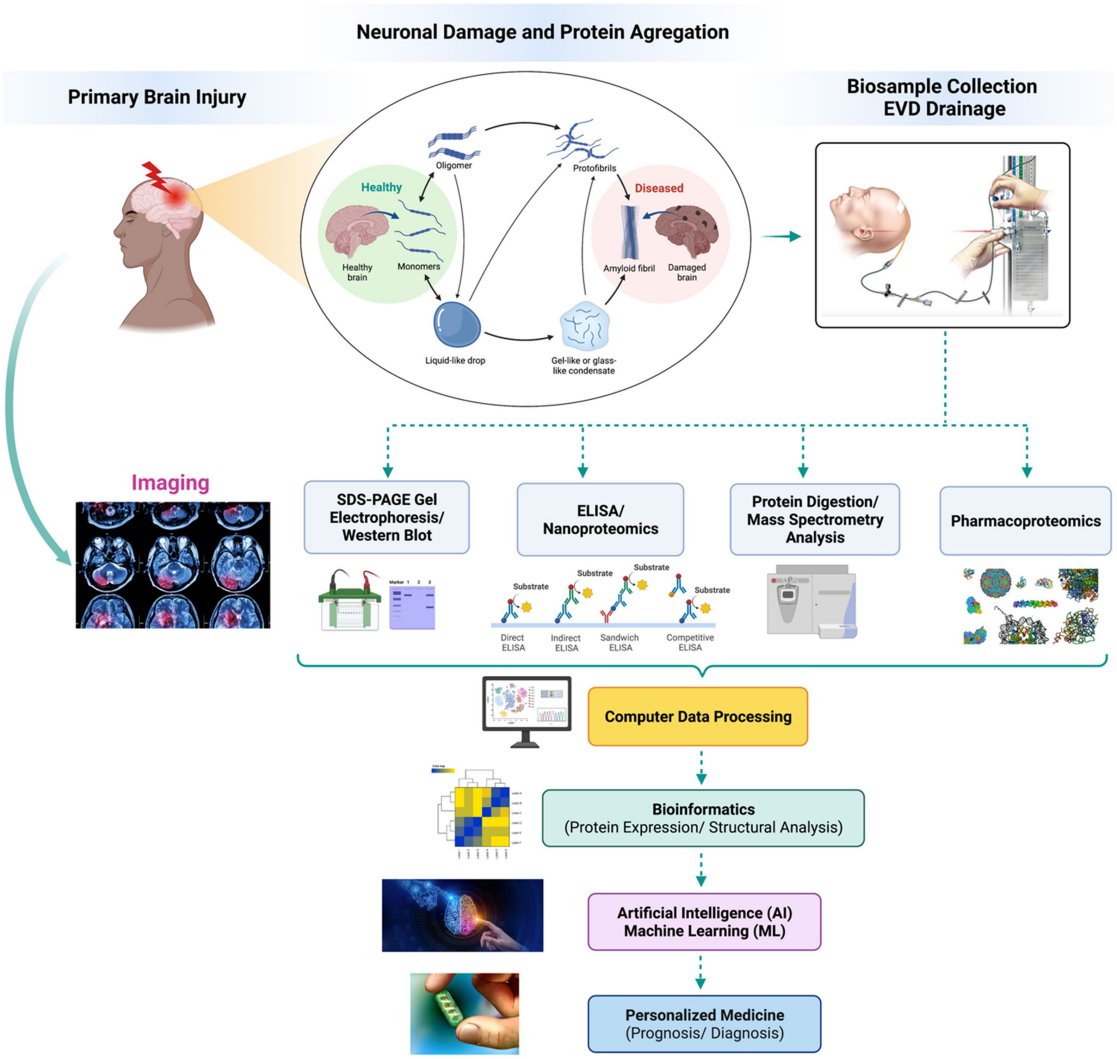
Proteomics, machine learning, and artificial intelligence in personalized medicine. In human clinical trials, neuroscientist study the proteomics of primary injury brain samples. After a primary injury, homeostatic conditions within the brain lead to kinase enzyme activation, PTM addition, and NFT formation, leading to plaque and malformation. Evidence of the damage was once primarily collected through neuroimaging, however faster and more effective technique have been developed through proteomics data analysis, computer data processing, AI and ML. Today, the collection of different data from a variety of tests provides significant data for special cases of TBI and can even provide better prognostic and diagnostic outputs for personalized medicine.

With various clinical studies, different quantification techniques, and diverse neurodegenerative outcomes, scientists have outperformed standard diagnosis matrices essential for the precision medicine (277, 282). Targeting treatments, molecular biology, and neuroproteomics approaches have led to novel diagnostic and treatment protocols. Researching various approaches, combining the data through ML/AI, and performing various proteomics studies will all be essential to developing these future advancements in clinical proteomics and precision medicine (273, 277, 286) (Figure 7).

## 5 Future directions and limitations

In the field of personalized medicine for neurotrauma, the implementation of neuroproteomics has emerged as a revolutionary approach. Neuroproteomics involves the large-scale study of proteins in the nervous system, allowing researchers to understand the intricate molecular mechanisms underlying neurotrauma at an individual level (290, 291). By analyzing the unique protein profiles in patients, clinicians can tailor treatments more precisely, optimizing therapeutic strategies for better outcomes. This personalized approach ensures that interventions are specifically designed to match the molecular intricacies of each patient’s conditions, leading to more effective and targeted treatments. Neuroproteomics, therefore, plays a pivotal role in advancing personalized medicine for neurotrauma by providing valuable insights into the diverse protein signatures associated with brain injuries, guiding clinicians toward a more informed decision-making (287, 292).

Moreover, the integration of AI and ML techniques significantly enhances the potential of neuroproteomics in personalized medicine (293). AI algorithms can process vast datasets, identify intricate patterns within protein profiles, and predict patient responses to different treatments (294). ML algorithms, on the other hand, can learn and adapt from these patterns, enabling real-time analysis and personalized treatment recommendations (295). This synergy between neuroproteomics and AI/ML technologies accelerates the pace of research, leading to more accurate diagnostic tools, targeted therapies, and predictive models (296). Looking ahead, the future of personalized medicine in neurotrauma lies in the continued advancement of these AI and ML applications, enabling rapid and precise decision-making based on a comprehensive molecular data (297). However, it is essential to acknowledge the limitations, including the need for large diverse datasets, ethical considerations, and the interpretability of AI-driven results (298). Addressing these challenges will be crucial for the seamless integration of neuroproteomics and AI/ML in personalized medicine, ensuring its transformative potential is fully realized.

It is also worth mentioning that although developing an effective tool in neuroscientific research, neuroproteomics and coupled technological advancements still have their limitations, human neuroproteomics research constraints lie in sample collection, subject availability, filtration, and identification (283).

First, finding potential human subjects and analyzing the impact of neurodegeneration on living humans is difficult due to the ethical standards of sample collection. Studying samples from live humans is extremely difficult due to the intravenous procedures necessary to conduct studies (284). Obtaining samples for proteomics research through intervention can be a complicated process. Most scientific data today rely on cadaver samples, EVD serum samples, animal models, and the unicellular proteomics (283, 284). While new methods of collecting samples are evaluated with studies like TRACK-TBI and CENTER-TBI, obtaining consent and sufficient samples for evaluation is still complicated due to the ethics behind obtaining informed consent, maintaining privacy, and attaining access to the rights of individual data. It is essential to tackle global data issues and create a global consensus on regulating privacy and consent among different political and healthcare systems (283).

Second, the collection of global human data is vast, and AI and ML would need to be updated to their most robust versions. With a problem holding extensive data, updated computer systems and robust tools will be essential for depositing and accessing necessary data. Innovators of such systems must be wary of automated backup and ensure the uniformity of data. All medical practices should also follow standard operating procedures of data collection, input, and analysis to maintain controlled and accurate diagnosis and the best medical practices worldwide.

In continuation, elaborating on the processes of sample purification, the filtration of proteins is challenging because many samples contain a mixture of different proteins and various impurities. Purification of protein is complex because contamination of nucleotides, peptides, lipids, and biofluids disrupts the isolation of specific protein structures. ELISA, western blot, mass spectrometry, protein sequencing, bioinformatics, and all the proteomic tests require pure protein filtration. Experimenting and identifying methods to purify specific proteins are being studied and continue to be a major advancement within proteomics. Basic protocols for purification methods, enzyme degradation, filtration, and treatment are continually evaluated in many studies (283, 299).

After sample purification, the last evaluation stage also has a few limitations. Bioinformatics, proteomics, and their respective databases are not all fully curated. Evaluation of bioinformatics databases and the use of technological prediction must always be carefully monitored. Support from multiple databases and experimentation is critical for supportive evidence. It is critical to utilize multiple tests, evaluation protocols, and various techniques in bioinformatics to create the most precise predictions and conclusions (283, 299).

Finally, although there are many limitations, the benefits of neuroproteomics-based personalized medicine outweigh the overall effects. Innovation and technological advancement in science will always require funding, and scientists must predict such endeavors’ costs. Conflicts may arise among multidisciplinary, multi-institutional, and multinational groups pursuing grants to support personalized medicine. Regardless of the conflicts and limitations, innovating such technologies and collaborating on a global scale will benefit the future of precision-based and personalized medicine.

## 6 Conclusion

Despite the drawbacks discussed, the field of proteomics is developing rapidly. In future endeavors, neuroscientists hope to find a fast, efficient, and significant *in-vitro*, non-intravenous protocol for neuroproteomics clinical diagnosis. Techniques like unicellular neuroproteomics have been coupled with AI and ML to identify the significant correlations and patterns within neuroproteomics data to accomplish these goals. Today, scientists are continuing their studies on what to improve in the subfield of neuroproteomics and how it can be utilized for neurodiagnostic, prognostic, and theragnostic protocols (258, 283).

Today different fields of unicellular proteomics are being utilized to conduct neuroproteomics studies. As described in many previous examples, unicellular proteomics focuses on specific cell types and analyzes the proteins that come from them (283). In many TBI-based studies, scientists evaluate the concentrations of cytokines, macrophages, antibodies, and other immuno-based proteins as potential indicators of neurodegenerative diseases (276, 284, 287, 288). Analyzing innate immune responses to foreign conglomerated proteins and experimenting with specific unicellular protein structures are all potential futures (274). Other factors are analyzed, like the effect of time, secondary injuries, and the concentrations of immunological biomarkers, all essential data for effective clinical prediction models (258, 283, 300).

In continuation, AI and ML systems’ contribution has already been discussed but are also essential, as they will gather large sets of data and analyze them through the system biology (21). AI and ML will tie together proteomics data and carry out significant correlations with the knowledge built on by hundreds of studies. Understanding how to correlate proteomics trends and qualitative diagnostic scores through technological means will revolutionize medicine, especially in the neurology and neurotrauma (222, 227, 239, 258, 283).

In summary, novel processes and protocols continue progressing in neuroproteomics and biotechnology. New techniques and procedures will help spread new therapies, prognostic tools, diagnostic biotechnologies, and future insights for efficient, accurate, and more precise personalized medicine (258, 283, 299).

## 7 Expert opinion

The value of neuroproteomics as a powerful tool to identify proteins reflecting and involved with specific neuropathobiological processes in TBI has been demonstrated in recent years. Neuroproteomics characterization of biomarkers (i.e., UCH-L1 and GFAP) (185, 301–303) that are now, for the first time, approved by regulatory authorities to aid in the diagnosis of TBI and play a key role in clinical studies are notable examples (304–306). Nonetheless, the molecular complexity of the post-translational modifications (e.g., phosphorylation and glycosylation) and related impact on protein function, pathophysiological processes, and brain interaction networks remain largely unexplored (57, 74). This knowledge holds critical potential for understanding the underlying pathobiology of TBI, paving the way to discover new biomarkers that are also therapeutic targets. To this end, bioinformatic handling of the large volumes of data generated by high-throughput technologies coupled with AI and ML statistical approaches will be essential to meaningful interpret information with respect to the functional and mechanistic value as well as to disentangle data’s heterogeneity and reveal pathways, expression patterns, and phenotypes toward precision medicine.

Tremendous progress has been made in the technologies employed in proteomics studies (high-resolution LC/MS, protein arrays, chip-based technologies, and single-cell proteomics, among others). These tools have become available not only to laboratory researchers but also to clinical settings; however, the ultimate output is to deliver clinically useful point-of-care tests capable of sensitive, accurate, rapid, inexpensive, and reproducible assessment across large patient cohorts.

Importantly, the main problem associated with neuroproteomics in TBI is the inconsistencies and lack of reproducibility of research findings due to the variable quality of the studies and differences in methodology. Therefore, there is an urgent need to standardize methodologies and define rigorous protocols for the bioinformatic and advanced computational analyzes to magnify the impact of the neuroproteomics data. Finally, international multidisciplinary collaborations combining different and complementary expertise and involving physicians, scientists, and programmers may have an instrumental role in analyzing the data proteomics in TBI and developing an effective framework for successful clinical implementation.

## Author contributions

FK: Conceptualization, Visualization, Writing – original draft, Writing – review & editing. MG: Conceptualization, Visualization, Writing – original draft, Writing – review & editing. HY: Conceptualization, Visualization, Writing – original draft, Writing – review & editing. ZS: Writing – review & editing. MK: Writing – review & editing. MH: Writing – review & editing. SA-R: Writing – review & editing. SM: Writing – review & editing. KW: Writing – review & editing. YM: Conceptualization, Funding acquisition, Visualization, Writing – review & editing.
